# Supplementary material for: Aura-biomes are present in the water layer above coral reef benthic macro-organisms
Source: PeerJ. 2017 Aug 15;5:e3666. doi: 10.7717/peerj.3666 (PMC5562181; doi:10.7717/peerj.3666)
Supplement: Table S1 [file peerj-05-3666-s001.docx]

| Macro-Organism | Percent Cover | Average Percent | Standard Error (%) |
| --- | --- | --- | --- |
| Coral | 76.66 | 78.33 | 2.89 |
| Coral | 86.66 |  |  |
| Coral | 76.66 |  |  |
| Coral | 73.33 |  |  |
| Fleshy macro-algae | 93.33 | 95.55 | 1.11 |
| Fleshy macro-algae | 96.66 |  |  |
| Fleshy macro-algae | 96.66 |  |  |
| Turf algae | 93.33 | 86.66 | 3.85 |
| Turf algae | 80.00 |  |  |
| Turf algae | 86.66 |  |  |
| Zoanthid | 93.33 | 88.33 | 5.00 |
| Zoanthid | 83.33 |  |  |

Supplementary Table 1. The percent cover of each replicate benthic organism.
