# Supplementary material for: Aura-biomes are present in the water layer above coral reef benthic macro-organisms
Source: PeerJ. 2017 Aug 15;5:e3666. doi: 10.7717/peerj.3666 (PMC5562181; doi:10.7717/peerj.3666)
Supplement: Table S2 — One standard error is provided for reads and GC content. [file peerj-05-3666-s002.docx]

| Sample Name | MG-Rast ID | Number of Reads | Average Read (bp) | Average GC Content (%) | Predicted Protein Features |
| --- | --- | --- | --- | --- | --- |
|  |  |  |  |  |  |
| Coral 1 | 4618077.3 | 1,547,432 | 236 ± 76 | 45 ± 11 | 1,066,051 |
| Coral 2 | 4618078.3 | 1,442,208 | 250 ± 75 | 42 ± 11 | 1,016,798 |
| Coral 3 | 4618738.3 | 5,170,644 | 269 ± 84 | 42 ± 12 | 3,143,913 |
| Coral 4 | 4618079.3 | 218,222 | 295 ± 75 | 50 ± 8 | 178,919 |
| Fleshy macro-algae 1 | 4618073.3 | 416,664 | 294 ± 83 | 44 ± 11 | 336,734 |
| Fleshy macro-algae 2 | 4618074.3 | 163,799 | 310 ± 76 | 50 ± 10 | 162,264 |
| Fleshy macro-algae 3 | 4618075.3 | 935,734 | 309 ± 79 | 51 ± 10 | 880,019 |
| Turf algae 1 | 4618076.3 | 1,590,620 | 265 ± 105 | 54 ± 9 | 1,436,169 |
| Turf algae 2 | 4618080.3 | 1,577,440 | 265 ± 73 | 45 ± 11 | 1,196,647 |
| Turf algae 3 | 4618081.3 | 1,272,272 | 315 ± 89 | 49 ± 11 | 1,115,547 |
| Zoanthid 1 | 4618085.3 | 472,615 | 276 ± 85 | 42 ± 9 | 388,584 |
| Zoanthid 2 | 4618086.3 | 683,913 | 238 ± 77 | 41 ± 8 | 512,263 |
| Water Column 1 | 4618082.3 | 2,209,759 | 243 ± 76 | 44 ± 11 | 1,423,646 |
| Water Column 2 | 4618083.3 | 271,442 | 307 ± 80 | 45 ± 11 | 243,033 |
| Water Column 3 | 4618084.3 | 244,866 | 302 ± 81 | 49 ± 11 | 214,316 |
| Water Column 4 | 4618445.3 | 2,488,312 | 265 ± 101 | 50 ± 11 | 1,952,289 |

Supplementary Table 2. The summary statistics of the 16 metagenomes sequenced for the aura-biome analysis. One standard error is provided for reads and GC content.
