# Supplementary material for: Aura-biomes are present in the water layer above coral reef benthic macro-organisms
Source: PeerJ. 2017 Aug 15;5:e3666. doi: 10.7717/peerj.3666 (PMC5562181; doi:10.7717/peerj.3666)
Supplement: Table S3 — The proportion of sequences in the top 100 genera found in each family in each metagenome, Mb, Mussismilia braziliensis; Fma, fleshy macroalgae; TA, turf algae; Pc, Palythoa caribaeorum and WC, water column. [file peerj-05-3666-s003.docx]

Supplementary Table 3. The proportion of sequences in the top 100 genera found in each family in each metagenome, Co = Coral*,* Fma = fleshy macroalgae, TA = turf algae, Zo = Zoanthid and WC = water column.

| phylum | class | family | genus | Co1 | Co2 | Co3 | Co4 | Fma1 | Fma2 | Fma3 | TA1 | TA2 | TA3 | Zo1 | Zo2 | WC1 | WC2 | WC3 | WC4 |
| --- | --- | --- | --- | --- | --- | --- | --- | --- | --- | --- | --- | --- | --- | --- | --- | --- | --- | --- | --- |
| Acidobacteria | Solibacteres | Solibacteraceae | Candidatus Solibacter | 0.10 | 0.09 | 0.08 | 0.28 | 0.09 | 0.29 | 0.61 | 1.05 | 0.11 | 0.31 | 0.11 | 0.04 | 0.08 | 0.16 | 0.10 | 0.37 |
|  | unclassified (derived from Acidobacteria) | unclassified (derived from Acidobacteria) | Candidatus Koribacter | 0.06 | 0.06 | 0.04 | 0.10 | 0.05 | 0.14 | 0.25 | 0.36 | 0.05 | 0.12 | 0.06 | 0.04 | 0.05 | 0.07 | 0.06 | 0.15 |
| Actinobacteria | Actinobacteria (class) | Acidothermaceae | Acidothermus | 0.02 | 0.03 | 0.02 | 0.06 | 0.02 | 0.06 | 0.06 | 0.13 | 0.02 | 0.04 | 0.01 | 0.02 | 0.02 | 0.03 | 0.03 | 0.04 |
|  |  | Bifidobacteriaceae | Bifidobacterium | 0.01 | 0.02 | 0.02 | 0.04 | 0.02 | 0.02 | 0.03 | 0.03 | 0.01 | 0.02 | 0.02 | 0.01 | 0.02 | 0.02 | 0.03 | 0.02 |
|  |  | Brevibacteriaceae | Brevibacterium | 0.01 | 0.01 | 0.02 | 0.06 | 0.02 | 0.03 | 0.03 | 0.03 | 0.01 | 0.02 | 0.01 | 0.02 | 0.01 | 0.02 | 0.02 | 0.02 |
|  |  | Catenulisporaceae | Catenulispora | 0.03 | 0.03 | 0.03 | 0.09 | 0.03 | 0.07 | 0.10 | 0.13 | 0.04 | 0.06 | 0.05 | 0.04 | 0.03 | 0.04 | 0.04 | 0.06 |
|  |  | Cellulomonadaceae | Cellulomonas | 0.01 | 0.01 | 0.01 | 0.06 | 0.01 | 0.03 | 0.03 | 0.06 | 0.01 | 0.02 | 0.01 | 0.01 | 0.01 | 0.01 | 0.02 | 0.02 |
|  |  | Conexibacteraceae | Conexibacter | 0.04 | 0.03 | 0.04 | 0.10 | 0.04 | 0.07 | 0.14 | 0.17 | 0.03 | 0.07 | 0.05 | 0.03 | 0.03 | 0.05 | 0.06 | 0.08 |
|  |  | Coriobacteriaceae | Atopobium | 0.00 | 0.01 | 0.00 | 0.01 | 0.01 | 0.01 | 0.01 | 0.01 | 0.01 | 0.01 | 0.01 | 0.00 | 0.00 | 0.01 | 0.00 | 0.01 |
|  |  |  | Cryptobacterium | 0.01 | 0.00 | 0.01 | 0.01 | 0.00 | 0.01 | 0.01 | 0.01 | 0.00 | 0.01 | 0.01 | 0.00 | 0.01 | 0.01 | 0.01 | 0.01 |
|  |  |  | Eggerthella | 0.01 | 0.01 | 0.01 | 0.02 | 0.01 | 0.02 | 0.02 | 0.02 | 0.01 | 0.01 | 0.01 | 0.01 | 0.01 | 0.01 | 0.01 | 0.01 |
|  |  |  | Slackia | 0.01 | 0.01 | 0.01 | 0.02 | 0.01 | 0.03 | 0.02 | 0.01 | 0.01 | 0.02 | 0.01 | 0.01 | 0.01 | 0.01 | 0.01 | 0.01 |
|  |  | Corynebacteriaceae | Corynebacterium | 0.04 | 0.05 | 0.04 | 0.15 | 0.06 | 0.07 | 0.07 | 0.09 | 0.04 | 0.05 | 0.05 | 0.04 | 0.04 | 0.05 | 0.06 | 0.06 |
|  |  | Dermabacteraceae | Brachybacterium | 0.01 | 0.01 | 0.01 | 0.06 | 0.01 | 0.02 | 0.03 | 0.04 | 0.01 | 0.01 | 0.01 | 0.02 | 0.01 | 0.02 | 0.02 | 0.01 |
|  |  | Dermacoccaceae | Kytococcus | 0.01 | 0.01 | 0.01 | 0.04 | 0.01 | 0.02 | 0.02 | 0.04 | 0.01 | 0.02 | 0.01 | 0.01 | 0.01 | 0.02 | 0.02 | 0.01 |
|  |  | Frankiaceae | Frankia | 0.08 | 0.09 | 0.08 | 0.26 | 0.08 | 0.24 | 0.29 | 0.41 | 0.09 | 0.18 | 0.08 | 0.05 | 0.07 | 0.12 | 0.12 | 0.18 |
|  |  | Geodermatophilaceae | Geodermatophilus | 0.02 | 0.02 | 0.03 | 0.12 | 0.03 | 0.07 | 0.07 | 0.12 | 0.03 | 0.06 | 0.03 | 0.01 | 0.02 | 0.04 | 0.02 | 0.05 |
|  |  | Glycomycetaceae | Stackebrandtia | 0.02 | 0.02 | 0.02 | 0.03 | 0.02 | 0.05 | 0.06 | 0.10 | 0.02 | 0.04 | 0.03 | 0.01 | 0.02 | 0.04 | 0.02 | 0.04 |
|  |  | Gordoniaceae | Gordonia | 0.02 | 0.01 | 0.02 | 0.07 | 0.02 | 0.04 | 0.05 | 0.05 | 0.02 | 0.03 | 0.03 | 0.02 | 0.01 | 0.03 | 0.03 | 0.03 |
|  |  | Intrasporangiaceae | Janibacter | 0.02 | 0.02 | 0.02 | 0.07 | 0.02 | 0.05 | 0.05 | 0.06 | 0.02 | 0.03 | 0.02 | 0.01 | 0.02 | 0.02 | 0.03 | 0.03 |
|  |  | Jonesiaceae | Jonesia | 0.01 | 0.01 | 0.01 | 0.03 | 0.01 | 0.01 | 0.02 | 0.02 | 0.01 | 0.01 | 0.01 | 0.01 | 0.01 | 0.01 | 0.01 | 0.01 |
|  |  | Kineosporiaceae | Kineococcus | 0.01 | 0.01 | 0.01 | 0.09 | 0.01 | 0.03 | 0.04 | 0.07 | 0.01 | 0.03 | 0.02 | 0.01 | 0.01 | 0.02 | 0.03 | 0.03 |
|  |  | Microbacteriaceae | Clavibacter | 0.01 | 0.01 | 0.01 | 0.02 | 0.01 | 0.03 | 0.02 | 0.04 | 0.01 | 0.01 | 0.01 | 0.00 | 0.01 | 0.01 | 0.02 | 0.01 |
|  |  |  | Leifsonia | 0.01 | 0.01 | 0.01 | 0.03 | 0.00 | 0.01 | 0.02 | 0.03 | 0.01 | 0.01 | 0.01 | 0.01 | 0.01 | 0.00 | 0.01 | 0.01 |
|  |  | Micrococcaceae | Arthrobacter | 0.00 | 0.00 | 0.00 | 0.01 | 0.00 | 0.00 | 0.00 | 0.01 | 0.00 | 0.00 | 0.00 | 0.00 | 0.00 | 0.00 | 0.00 | 0.00 |
|  |  |  | Kocuria | 0.01 | 0.01 | 0.01 | 0.15 | 0.01 | 0.02 | 0.02 | 0.04 | 0.01 | 0.02 | 0.01 | 0.01 | 0.01 | 0.01 | 0.02 | 0.01 |
|  |  |  | Renibacterium | 0.01 | 0.01 | 0.01 | 0.03 | 0.01 | 0.02 | 0.02 | 0.02 | 0.01 | 0.01 | 0.01 | 0.01 | 0.01 | 0.01 | 0.02 | 0.01 |
|  |  | Micromonosporaceae | Salinispora | 0.05 | 0.06 | 0.06 | 0.13 | 0.04 | 0.11 | 0.14 | 0.20 | 0.05 | 0.08 | 0.04 | 0.03 | 0.06 | 0.07 | 0.07 | 0.09 |
|  |  | Mycobacteriaceae | Mycobacterium | 0.15 | 0.15 | 0.13 | 0.43 | 0.18 | 0.45 | 0.57 | 0.66 | 0.16 | 0.29 | 0.23 | 0.19 | 0.14 | 0.17 | 0.21 | 0.34 |
|  |  | Nakamurellaceae | Nakamurella | 0.02 | 0.02 | 0.02 | 0.10 | 0.02 | 0.05 | 0.06 | 0.10 | 0.02 | 0.04 | 0.03 | 0.02 | 0.02 | 0.02 | 0.02 | 0.04 |
|  |  | Nocardiaceae | Nocardia | 0.03 | 0.03 | 0.03 | 0.07 | 0.02 | 0.08 | 0.08 | 0.10 | 0.03 | 0.05 | 0.03 | 0.02 | 0.03 | 0.04 | 0.02 | 0.05 |
|  |  |  | Rhodococcus | 0.04 | 0.04 | 0.04 | 0.12 | 0.04 | 0.09 | 0.13 | 0.16 | 0.05 | 0.09 | 0.04 | 0.02 | 0.04 | 0.04 | 0.06 | 0.09 |
|  |  | Nocardioidaceae | Kribbella | 0.02 | 0.03 | 0.03 | 0.11 | 0.04 | 0.07 | 0.08 | 0.12 | 0.03 | 0.05 | 0.03 | 0.02 | 0.03 | 0.04 | 0.04 | 0.05 |
|  |  |  | Nocardioides | 0.03 | 0.03 | 0.03 | 0.16 | 0.04 | 0.09 | 0.12 | 0.17 | 0.03 | 0.08 | 0.03 | 0.01 | 0.03 | 0.04 | 0.05 | 0.08 |
|  |  | Nocardiopsaceae | Nocardiopsis | 0.02 | 0.02 | 0.02 | 0.06 | 0.03 | 0.05 | 0.05 | 0.08 | 0.02 | 0.04 | 0.03 | 0.02 | 0.02 | 0.03 | 0.02 | 0.03 |
|  |  |  | Thermobifida | 0.02 | 0.03 | 0.02 | 0.05 | 0.02 | 0.04 | 0.06 | 0.11 | 0.02 | 0.05 | 0.02 | 0.01 | 0.02 | 0.04 | 0.04 | 0.05 |
|  |  | Promicromonosporaceae | Xylanimonas | 0.01 | 0.01 | 0.01 | 0.02 | 0.01 | 0.02 | 0.02 | 0.04 | 0.01 | 0.02 | 0.01 | 0.00 | 0.01 | 0.02 | 0.01 | 0.01 |
|  |  | Propionibacteriaceae | Propionibacterium | 0.03 | 0.02 | 0.02 | 0.09 | 0.01 | 0.02 | 0.02 | 0.03 | 0.01 | 0.02 | 0.02 | 0.03 | 0.01 | 0.02 | 0.06 | 0.02 |
|  |  | Pseudonocardiaceae | Actinosynnema | 0.02 | 0.02 | 0.02 | 0.07 | 0.03 | 0.05 | 0.06 | 0.09 | 0.02 | 0.04 | 0.02 | 0.01 | 0.02 | 0.04 | 0.03 | 0.04 |
|  |  |  | Saccharomonospora | 0.02 | 0.02 | 0.02 | 0.05 | 0.03 | 0.05 | 0.06 | 0.07 | 0.02 | 0.03 | 0.02 | 0.02 | 0.02 | 0.02 | 0.03 | 0.04 |
|  |  |  | Saccharopolyspora | 0.03 | 0.03 | 0.02 | 0.08 | 0.03 | 0.06 | 0.09 | 0.13 | 0.03 | 0.06 | 0.02 | 0.02 | 0.03 | 0.04 | 0.05 | 0.06 |
|  |  |  | Thermobispora | 0.02 | 0.03 | 0.02 | 0.07 | 0.03 | 0.06 | 0.07 | 0.10 | 0.02 | 0.04 | 0.04 | 0.02 | 0.03 | 0.04 | 0.04 | 0.04 |
|  |  | Rubrobacteraceae | Rubrobacter | 0.04 | 0.05 | 0.03 | 0.14 | 0.04 | 0.08 | 0.12 | 0.21 | 0.04 | 0.08 | 0.04 | 0.04 | 0.05 | 0.04 | 0.07 | 0.09 |
|  |  | Streptomycetaceae | Streptomyces | 0.10 | 0.11 | 0.09 | 0.29 | 0.10 | 0.21 | 0.30 | 0.44 | 0.10 | 0.19 | 0.11 | 0.08 | 0.09 | 0.12 | 0.15 | 0.19 |
|  |  | Streptosporangiaceae | Streptosporangium | 0.03 | 0.03 | 0.03 | 0.09 | 0.04 | 0.09 | 0.13 | 0.18 | 0.04 | 0.08 | 0.04 | 0.03 | 0.03 | 0.04 | 0.06 | 0.07 |
|  |  | Thermomonosporaceae | Thermomonospora | 0.04 | 0.03 | 0.04 | 0.10 | 0.03 | 0.10 | 0.11 | 0.13 | 0.03 | 0.07 | 0.03 | 0.03 | 0.03 | 0.05 | 0.06 | 0.07 |
|  |  | Tsukamurellaceae | Tsukamurella | 0.01 | 0.01 | 0.01 | 0.05 | 0.02 | 0.04 | 0.04 | 0.04 | 0.01 | 0.02 | 0.03 | 0.01 | 0.01 | 0.01 | 0.02 | 0.02 |
|  |  | unclassified (derived from Actinobacteria (class)) | unclassified (derived from Actinobacteria (class)) | 0.01 | 0.01 | 0.02 | 0.02 | 0.02 | 0.02 | 0.02 | 0.03 | 0.01 | 0.02 | 0.02 | 0.01 | 0.01 | 0.02 | 0.03 | 0.02 |
|  |  | unclassified (derived from Actinomycetales) | Tropheryma | 0.00 | 0.01 | 0.01 | 0.01 | 0.01 | 0.01 | 0.00 | 0.00 | 0.00 | 0.00 | 0.00 | 0.00 | 0.00 | 0.00 | 0.01 | 0.00 |
| Aquificae | Aquificae (class) | Aquificaceae | Aquifex | 0.03 | 0.04 | 0.03 | 0.02 | 0.03 | 0.02 | 0.05 | 0.06 | 0.02 | 0.03 | 0.02 | 0.01 | 0.03 | 0.02 | 0.02 | 0.03 |
|  |  |  | Hydrogenobaculum | 0.01 | 0.01 | 0.01 | 0.00 | 0.01 | 0.01 | 0.01 | 0.01 | 0.01 | 0.01 | 0.01 | 0.01 | 0.01 | 0.01 | 0.01 | 0.01 |
|  |  | Hydrogenothermaceae | Sulfurihydrogenibium | 0.02 | 0.03 | 0.03 | 0.03 | 0.03 | 0.02 | 0.03 | 0.04 | 0.01 | 0.02 | 0.02 | 0.02 | 0.02 | 0.03 | 0.02 | 0.02 |
| Bacteroidetes | Bacteroidia | Bacteroidaceae | Bacteroides | 0.21 | 0.25 | 0.25 | 0.38 | 0.20 | 0.43 | 0.43 | 0.46 | 0.22 | 0.38 | 0.21 | 0.12 | 0.25 | 0.24 | 0.34 | 0.32 |
|  |  | Porphyromonadaceae | Parabacteroides | 0.10 | 0.12 | 0.11 | 0.13 | 0.10 | 0.21 | 0.23 | 0.28 | 0.11 | 0.21 | 0.10 | 0.05 | 0.13 | 0.13 | 0.15 | 0.17 |
|  |  |  | Porphyromonas | 0.06 | 0.07 | 0.07 | 0.07 | 0.06 | 0.10 | 0.11 | 0.10 | 0.07 | 0.10 | 0.06 | 0.03 | 0.08 | 0.07 | 0.09 | 0.09 |
|  | Cytophagia | Cytophagaceae | Cytophaga | 0.27 | 0.32 | 0.31 | 0.21 | 0.27 | 0.59 | 0.81 | 0.61 | 0.30 | 0.66 | 0.22 | 0.12 | 0.33 | 0.37 | 0.38 | 0.49 |
|  |  |  | Dyadobacter | 0.19 | 0.19 | 0.18 | 0.29 | 0.20 | 0.47 | 0.54 | 0.56 | 0.20 | 0.43 | 0.19 | 0.08 | 0.20 | 0.25 | 0.33 | 0.33 |
|  |  |  | Spirosoma | 0.23 | 0.22 | 0.22 | 0.38 | 0.23 | 0.55 | 0.67 | 0.61 | 0.23 | 0.49 | 0.24 | 0.13 | 0.23 | 0.29 | 0.32 | 0.39 |
|  | Flavobacteriia | Flavobacteriaceae | Capnocytophaga | 0.00 | 0.00 | 0.00 | 0.00 | 0.00 | 0.00 | 0.00 | 0.00 | 0.00 | 0.00 | 0.00 | 0.00 | 0.00 | 0.00 | 0.00 | 0.00 |
|  |  |  | Croceibacter | 0.77 | 0.75 | 0.90 | 1.07 | 0.72 | 1.07 | 0.81 | 0.74 | 0.72 | 1.08 | 0.72 | 0.31 | 0.82 | 0.78 | 1.39 | 0.66 |
|  |  |  | Dokdonia | 0.49 | 0.52 | 0.63 | 0.86 | 0.49 | 0.89 | 0.69 | 0.61 | 0.54 | 0.82 | 0.52 | 0.24 | 0.59 | 0.58 | 0.97 | 0.51 |
|  |  |  | Flavobacterium | 0.55 | 0.71 | 0.79 | 0.82 | 0.55 | 0.65 | 0.58 | 0.50 | 0.60 | 0.80 | 0.43 | 0.21 | 0.69 | 0.59 | 0.97 | 0.52 |
|  |  |  | Gramella | 1.78 | 1.78 | 2.03 | 2.02 | 1.44 | 1.84 | 1.69 | 1.45 | 1.61 | 2.34 | 1.27 | 0.58 | 1.86 | 1.56 | 2.80 | 1.46 |
|  |  |  | Leeuwenhoekiella | 0.59 | 0.58 | 0.69 | 0.98 | 0.53 | 0.93 | 0.64 | 0.55 | 0.54 | 1.10 | 0.60 | 0.25 | 0.60 | 0.65 | 1.27 | 0.51 |
|  |  |  | Maribacter | 1.12 | 1.19 | 1.42 | 1.94 | 1.14 | 1.82 | 1.49 | 1.31 | 1.17 | 1.70 | 1.11 | 0.53 | 1.28 | 1.42 | 2.43 | 1.10 |
|  |  |  | Polaribacter | 0.94 | 0.96 | 1.18 | 1.89 | 0.90 | 1.62 | 1.20 | 0.99 | 0.92 | 1.36 | 1.14 | 0.40 | 1.49 | 1.13 | 1.85 | 0.94 |
|  |  |  | Riemerella | 0.00 | 0.00 | 0.00 | 0.00 | 0.00 | 0.00 | 0.00 | 0.00 | 0.00 | 0.00 | 0.00 | 0.00 | 0.00 | 0.00 | 0.00 | 0.00 |
|  |  |  | Robiginitalea | 0.73 | 0.80 | 0.95 | 1.34 | 0.74 | 1.27 | 0.94 | 0.80 | 0.76 | 1.13 | 0.74 | 0.36 | 0.86 | 0.88 | 1.57 | 0.76 |
|  | Sphingobacteriia | Sphingobacteriaceae | Pedobacter | 0.20 | 0.23 | 0.24 | 0.24 | 0.24 | 0.50 | 0.51 | 0.43 | 0.22 | 0.39 | 0.21 | 0.11 | 0.22 | 0.27 | 0.39 | 0.33 |
|  |  | unclassified (derived from Sphingobacteriales) | Chitinophaga | 0.22 | 0.21 | 0.21 | 0.25 | 0.23 | 0.45 | 0.57 | 0.45 | 0.21 | 0.43 | 0.19 | 0.09 | 0.22 | 0.29 | 0.34 | 0.34 |
|  | unclassified (derived from Bacteroidetes) | Rhodothermaceae | Rhodothermus | 0.09 | 0.10 | 0.08 | 0.16 | 0.10 | 0.23 | 0.37 | 0.41 | 0.10 | 0.21 | 0.10 | 0.06 | 0.10 | 0.13 | 0.10 | 0.23 |
|  |  |  | Salinibacter | 0.07 | 0.07 | 0.05 | 0.12 | 0.06 | 0.12 | 0.22 | 0.24 | 0.07 | 0.13 | 0.05 | 0.04 | 0.07 | 0.08 | 0.08 | 0.14 |
| Chlamydiae | Chlamydiia | Chlamydiaceae | Chlamydia | 0.01 | 0.01 | 0.01 | 0.02 | 0.02 | 0.03 | 0.03 | 0.06 | 0.01 | 0.02 | 0.01 | 0.01 | 0.02 | 0.02 | 0.02 | 0.02 |
|  |  |  | Chlamydophila | 0.01 | 0.01 | 0.01 | 0.01 | 0.01 | 0.01 | 0.02 | 0.03 | 0.01 | 0.01 | 0.01 | 0.01 | 0.01 | 0.01 | 0.00 | 0.01 |
|  |  | Parachlamydiaceae | Candidatus Protochlamydia | 0.05 | 0.04 | 0.04 | 0.06 | 0.06 | 0.08 | 0.10 | 0.18 | 0.04 | 0.07 | 0.05 | 0.03 | 0.05 | 0.05 | 0.03 | 0.07 |
| Chlorobi | Chlorobia | Chlorobiaceae | Chlorobaculum | 0.03 | 0.04 | 0.03 | 0.06 | 0.04 | 0.06 | 0.08 | 0.10 | 0.03 | 0.06 | 0.03 | 0.03 | 0.04 | 0.05 | 0.05 | 0.05 |
|  |  |  | Chlorobium | 0.10 | 0.13 | 0.11 | 0.16 | 0.13 | 0.22 | 0.25 | 0.31 | 0.10 | 0.19 | 0.11 | 0.07 | 0.11 | 0.12 | 0.13 | 0.17 |
|  |  |  | Chloroherpeton | 0.04 | 0.05 | 0.04 | 0.07 | 0.05 | 0.08 | 0.13 | 0.14 | 0.04 | 0.08 | 0.04 | 0.05 | 0.04 | 0.07 | 0.05 | 0.08 |
|  |  |  | Pelodictyon | 0.04 | 0.04 | 0.03 | 0.05 | 0.03 | 0.05 | 0.09 | 0.10 | 0.03 | 0.06 | 0.04 | 0.02 | 0.03 | 0.05 | 0.05 | 0.05 |
|  |  |  | Prosthecochloris | 0.00 | 0.00 | 0.00 | 0.00 | 0.00 | 0.00 | 0.00 | 0.00 | 0.00 | 0.00 | 0.00 | 0.00 | 0.00 | 0.00 | 0.00 | 0.00 |
| Chloroflexi | Chloroflexi (class) | Chloroflexaceae | Chloroflexus | 0.06 | 0.06 | 0.05 | 0.13 | 0.06 | 0.14 | 0.18 | 0.27 | 0.05 | 0.12 | 0.05 | 0.04 | 0.06 | 0.07 | 0.07 | 0.11 |
|  |  |  | Roseiflexus | 0.06 | 0.05 | 0.04 | 0.11 | 0.06 | 0.10 | 0.17 | 0.27 | 0.05 | 0.10 | 0.05 | 0.03 | 0.05 | 0.06 | 0.05 | 0.12 |
|  |  | Herpetosiphonaceae | Herpetosiphon | 0.04 | 0.04 | 0.04 | 0.13 | 0.05 | 0.12 | 0.13 | 0.17 | 0.05 | 0.09 | 0.06 | 0.06 | 0.05 | 0.07 | 0.06 | 0.09 |
|  | Dehalococcoidetes | unclassified (derived from Dehalococcoidetes) | Dehalococcoides | 0.02 | 0.02 | 0.02 | 0.03 | 0.03 | 0.03 | 0.04 | 0.06 | 0.02 | 0.03 | 0.03 | 0.00 | 0.02 | 0.02 | 0.03 | 0.03 |
|  | Thermomicrobia (class) | Sphaerobacteraceae | Sphaerobacter | 0.04 | 0.04 | 0.04 | 0.09 | 0.04 | 0.10 | 0.15 | 0.19 | 0.04 | 0.08 | 0.03 | 0.03 | 0.04 | 0.07 | 0.08 | 0.08 |
|  |  | Thermomicrobiaceae | Thermomicrobium | 0.00 | 0.01 | 0.01 | 0.02 | 0.01 | 0.01 | 0.02 | 0.03 | 0.01 | 0.01 | 0.00 | 0.01 | 0.01 | 0.01 | 0.01 | 0.01 |
| Cyanobacteria | Gloeobacteria | unclassified (derived from Gloeobacterales) | Gloeobacter | 0.05 | 0.05 | 0.03 | 0.09 | 0.04 | 0.10 | 0.13 | 0.20 | 0.04 | 0.09 | 0.05 | 0.03 | 0.05 | 0.06 | 0.06 | 0.10 |
|  | unclassified (derived from Cyanobacteria) | Nostocaceae | Anabaena | 0.06 | 0.07 | 0.05 | 0.16 | 0.06 | 0.18 | 0.14 | 0.19 | 0.06 | 0.13 | 0.09 | 0.04 | 0.06 | 0.07 | 0.08 | 0.11 |
|  |  |  | Nostoc | 0.13 | 0.14 | 0.09 | 0.33 | 0.11 | 0.31 | 0.26 | 0.36 | 0.12 | 0.23 | 0.19 | 0.12 | 0.11 | 0.15 | 0.16 | 0.19 |
|  |  | Prochlorococcaceae | Prochlorococcus | 0.24 | 0.38 | 0.37 | 0.64 | 0.32 | 0.31 | 0.22 | 0.19 | 0.25 | 0.28 | 0.24 | 0.21 | 0.26 | 0.38 | 0.73 | 0.30 |
|  |  | unclassified (derived from Chroococcales) | Acaryochloris | 0.01 | 0.01 | 0.01 | 0.03 | 0.01 | 0.04 | 0.04 | 0.05 | 0.02 | 0.03 | 0.02 | 0.01 | 0.01 | 0.02 | 0.01 | 0.02 |
|  |  |  | Crocosphaera | 0.03 | 0.03 | 0.03 | 0.08 | 0.04 | 0.05 | 0.06 | 0.08 | 0.03 | 0.05 | 0.05 | 0.03 | 0.03 | 0.04 | 0.03 | 0.04 |
|  |  |  | Cyanothece | 0.04 | 0.04 | 0.04 | 0.12 | 0.04 | 0.09 | 0.10 | 0.12 | 0.04 | 0.07 | 0.06 | 0.04 | 0.04 | 0.06 | 0.07 | 0.06 |
|  |  |  | Microcystis | 0.04 | 0.03 | 0.03 | 0.13 | 0.04 | 0.09 | 0.08 | 0.09 | 0.03 | 0.05 | 0.06 | 0.03 | 0.02 | 0.03 | 0.06 | 0.05 |
|  |  |  | Synechococcus | 5.84 | 10.91 | 11.09 | 32.86 | 7.09 | 11.20 | 4.51 | 3.50 | 6.33 | 7.28 | 5.65 | 5.36 | 5.58 | 11.83 | 31.75 | 8.07 |
|  |  |  | Synechocystis | 0.05 | 0.09 | 0.03 | 0.09 | 0.05 | 0.09 | 0.11 | 0.14 | 0.05 | 0.09 | 0.06 | 0.05 | 0.07 | 0.05 | 0.06 | 0.09 |
|  |  |  | Thermosynechococcus | 0.04 | 0.06 | 0.03 | 0.09 | 0.05 | 0.08 | 0.07 | 0.08 | 0.03 | 0.06 | 0.04 | 0.04 | 0.04 | 0.05 | 0.10 | 0.07 |
|  |  | unclassified (derived from Oscillatoriales) | Trichodesmium | 0.10 | 0.13 | 0.10 | 0.20 | 0.12 | 0.17 | 0.14 | 0.18 | 0.08 | 0.15 | 0.14 | 0.09 | 0.11 | 0.09 | 0.08 | 0.13 |
| Deferribacteres | Deferribacteres (class) | Deferribacteraceae | Denitrovibrio | 0.04 | 0.05 | 0.04 | 0.02 | 0.05 | 0.05 | 0.06 | 0.07 | 0.03 | 0.04 | 0.04 | 0.03 | 0.04 | 0.04 | 0.03 | 0.05 |
| Deinococcus-Thermus | Deinococci | Deinococcaceae | Deinococcus | 0.05 | 0.04 | 0.04 | 0.08 | 0.04 | 0.09 | 0.11 | 0.14 | 0.04 | 0.07 | 0.04 | 0.03 | 0.05 | 0.05 | 0.04 | 0.08 |
|  |  | Thermaceae | Meiothermus | 0.05 | 0.06 | 0.04 | 0.07 | 0.06 | 0.12 | 0.13 | 0.17 | 0.04 | 0.08 | 0.05 | 0.03 | 0.05 | 0.07 | 0.06 | 0.09 |
|  |  |  | Thermus | 0.02 | 0.03 | 0.02 | 0.03 | 0.04 | 0.03 | 0.06 | 0.09 | 0.02 | 0.03 | 0.02 | 0.02 | 0.04 | 0.03 | 0.02 | 0.05 |
| Dictyoglomi | Dictyoglomia | Dictyoglomaceae | Dictyoglomus | 0.02 | 0.03 | 0.02 | 0.03 | 0.04 | 0.05 | 0.04 | 0.05 | 0.02 | 0.03 | 0.04 | 0.02 | 0.03 | 0.05 | 0.04 | 0.03 |
| Elusimicrobia | Elusimicrobia (class) | Elusimicrobiaceae | Elusimicrobium | 0.01 | 0.01 | 0.01 | 0.00 | 0.02 | 0.02 | 0.02 | 0.02 | 0.01 | 0.01 | 0.01 | 0.01 | 0.01 | 0.01 | 0.01 | 0.01 |
| Firmicutes | Bacilli | Alicyclobacillaceae | Alicyclobacillus | 0.02 | 0.02 | 0.01 | 0.03 | 0.02 | 0.06 | 0.04 | 0.06 | 0.01 | 0.03 | 0.02 | 0.02 | 0.02 | 0.02 | 0.02 | 0.03 |
|  |  | Bacillaceae | Anoxybacillus | 0.01 | 0.02 | 0.01 | 0.03 | 0.03 | 0.03 | 0.03 | 0.04 | 0.02 | 0.02 | 0.03 | 0.01 | 0.02 | 0.02 | 0.01 | 0.02 |
|  |  |  | Bacillus | 0.19 | 0.23 | 0.17 | 0.41 | 0.23 | 0.34 | 0.38 | 0.43 | 0.17 | 0.29 | 0.21 | 0.13 | 0.20 | 0.26 | 0.25 | 0.24 |
|  |  |  | Geobacillus | 0.05 | 0.05 | 0.04 | 0.07 | 0.06 | 0.07 | 0.10 | 0.11 | 0.03 | 0.06 | 0.04 | 0.04 | 0.05 | 0.05 | 0.06 | 0.06 |
|  |  |  | Lysinibacillus | 0.00 | 0.00 | 0.00 | 0.00 | 0.00 | 0.00 | 0.00 | 0.00 | 0.00 | 0.00 | 0.00 | 0.00 | 0.00 | 0.00 | 0.00 | 0.00 |
|  |  |  | Marinococcus | 0.00 | 0.00 | 0.00 | 0.00 | 0.00 | 0.00 | 0.00 | 0.00 | 0.00 | 0.00 | 0.00 | 0.00 | 0.00 | 0.00 | 0.00 | 0.00 |
|  |  |  | Oceanobacillus | 0.03 | 0.03 | 0.02 | 0.03 | 0.03 | 0.04 | 0.04 | 0.05 | 0.02 | 0.03 | 0.03 | 0.02 | 0.03 | 0.03 | 0.03 | 0.03 |
|  |  | Enterococcaceae | Enterococcus | 0.02 | 0.01 | 0.01 | 0.01 | 0.02 | 0.02 | 0.02 | 0.02 | 0.01 | 0.01 | 0.01 | 0.01 | 0.01 | 0.01 | 0.02 | 0.01 |
|  |  |  | Tetragenococcus | 0.00 | 0.00 | 0.00 | 0.00 | 0.00 | 0.00 | 0.00 | 0.00 | 0.00 | 0.00 | 0.00 | 0.00 | 0.00 | 0.00 | 0.00 | 0.00 |
|  |  | Lactobacillaceae | Lactobacillus | 0.04 | 0.04 | 0.04 | 0.03 | 0.05 | 0.04 | 0.07 | 0.07 | 0.03 | 0.04 | 0.04 | 0.04 | 0.04 | 0.05 | 0.05 | 0.04 |
|  |  |  | Pediococcus | 0.01 | 0.00 | 0.00 | 0.01 | 0.01 | 0.01 | 0.01 | 0.01 | 0.00 | 0.01 | 0.00 | 0.00 | 0.00 | 0.00 | 0.00 | 0.00 |
|  |  | Leuconostocaceae | Leuconostoc | 0.00 | 0.00 | 0.00 | 0.00 | 0.00 | 0.00 | 0.00 | 0.01 | 0.00 | 0.00 | 0.00 | 0.00 | 0.00 | 0.00 | 0.01 | 0.00 |
|  |  |  | Oenococcus | 0.00 | 0.01 | 0.00 | 0.01 | 0.00 | 0.00 | 0.01 | 0.01 | 0.00 | 0.01 | 0.01 | 0.01 | 0.00 | 0.00 | 0.00 | 0.00 |
|  |  | Listeriaceae | Listeria | 0.02 | 0.02 | 0.02 | 0.02 | 0.02 | 0.02 | 0.05 | 0.04 | 0.02 | 0.03 | 0.02 | 0.02 | 0.02 | 0.02 | 0.03 | 0.02 |
|  |  | Paenibacillaceae | Paenibacillus | 0.01 | 0.01 | 0.01 | 0.01 | 0.01 | 0.01 | 0.02 | 0.02 | 0.01 | 0.01 | 0.01 | 0.01 | 0.01 | 0.01 | 0.01 | 0.01 |
|  |  | Staphylococcaceae | Macrococcus | 0.00 | 0.00 | 0.00 | 0.00 | 0.00 | 0.00 | 0.00 | 0.00 | 0.00 | 0.00 | 0.00 | 0.00 | 0.00 | 0.00 | 0.00 | 0.00 |
|  |  |  | Staphylococcus | 0.06 | 0.04 | 0.05 | 0.74 | 0.06 | 0.10 | 0.05 | 0.06 | 0.07 | 0.05 | 0.05 | 0.09 | 0.04 | 0.05 | 0.06 | 0.03 |
|  |  | Streptococcaceae | Lactococcus | 0.01 | 0.01 | 0.01 | 0.02 | 0.01 | 0.02 | 0.01 | 0.02 | 0.01 | 0.01 | 0.01 | 0.01 | 0.01 | 0.01 | 0.00 | 0.01 |
|  |  |  | Streptococcus | 0.05 | 0.04 | 0.05 | 0.10 | 0.06 | 0.07 | 0.06 | 0.08 | 0.03 | 0.05 | 0.04 | 0.04 | 0.04 | 0.05 | 0.08 | 0.04 |
|  |  | unclassified (derived from Bacillales) | Exiguobacterium | 0.05 | 0.01 | 0.02 | 0.55 | 0.02 | 0.02 | 0.03 | 0.08 | 0.03 | 0.02 | 0.01 | 0.00 | 0.05 | 0.02 | 0.01 | 0.02 |
|  | Clostridia | Clostridiaceae | Alkaliphilus | 0.03 | 0.04 | 0.03 | 0.03 | 0.03 | 0.06 | 0.06 | 0.08 | 0.03 | 0.05 | 0.03 | 0.03 | 0.04 | 0.04 | 0.04 | 0.05 |
|  |  |  | Clostridium | 0.16 | 0.22 | 0.20 | 0.26 | 0.25 | 0.23 | 0.30 | 0.36 | 0.15 | 0.20 | 0.18 | 0.13 | 0.22 | 0.24 | 0.22 | 0.19 |
|  |  | Clostridiales Family XI. Incertae Sedis | Finegoldia | 0.00 | 0.00 | 0.00 | 0.00 | 0.00 | 0.00 | 0.00 | 0.00 | 0.00 | 0.00 | 0.00 | 0.00 | 0.00 | 0.00 | 0.00 | 0.00 |
|  |  | Clostridiales Family XVIII. Incertae Sedis | Symbiobacterium | 0.03 | 0.03 | 0.02 | 0.05 | 0.03 | 0.04 | 0.10 | 0.13 | 0.03 | 0.05 | 0.03 | 0.02 | 0.03 | 0.03 | 0.03 | 0.06 |
|  |  | Eubacteriaceae | Eubacterium | 0.01 | 0.01 | 0.01 | 0.02 | 0.01 | 0.03 | 0.02 | 0.02 | 0.01 | 0.01 | 0.01 | 0.02 | 0.01 | 0.02 | 0.01 | 0.01 |
|  |  | Halanaerobiaceae | Halothermothrix | 0.03 | 0.04 | 0.03 | 0.06 | 0.03 | 0.04 | 0.06 | 0.07 | 0.02 | 0.04 | 0.03 | 0.02 | 0.03 | 0.04 | 0.04 | 0.04 |
|  |  | Heliobacteriaceae | Heliobacterium | 0.01 | 0.02 | 0.02 | 0.03 | 0.02 | 0.04 | 0.04 | 0.06 | 0.01 | 0.03 | 0.02 | 0.01 | 0.02 | 0.03 | 0.03 | 0.03 |
|  |  | Natranaerobiaceae | Natranaerobius | 0.02 | 0.02 | 0.01 | 0.02 | 0.03 | 0.03 | 0.03 | 0.03 | 0.01 | 0.02 | 0.02 | 0.02 | 0.02 | 0.02 | 0.02 | 0.02 |
|  |  | Peptococcaceae | Desulfitobacterium | 0.02 | 0.03 | 0.02 | 0.02 | 0.03 | 0.06 | 0.06 | 0.09 | 0.02 | 0.04 | 0.03 | 0.03 | 0.02 | 0.04 | 0.05 | 0.04 |
|  |  |  | Desulfotomaculum | 0.03 | 0.04 | 0.03 | 0.06 | 0.04 | 0.07 | 0.08 | 0.12 | 0.03 | 0.06 | 0.04 | 0.03 | 0.03 | 0.06 | 0.06 | 0.06 |
|  |  |  | Pelotomaculum | 0.02 | 0.02 | 0.02 | 0.03 | 0.02 | 0.02 | 0.06 | 0.08 | 0.01 | 0.03 | 0.02 | 0.01 | 0.01 | 0.03 | 0.02 | 0.03 |
|  |  | Peptostreptococcaceae | unclassified (derived from Peptostreptococcaceae) | 0.01 | 0.02 | 0.02 | 0.02 | 0.02 | 0.03 | 0.03 | 0.03 | 0.02 | 0.02 | 0.03 | 0.01 | 0.02 | 0.03 | 0.03 | 0.02 |
|  |  | Syntrophomonadaceae | Syntrophomonas | 0.01 | 0.03 | 0.02 | 0.01 | 0.03 | 0.02 | 0.04 | 0.05 | 0.02 | 0.02 | 0.02 | 0.01 | 0.02 | 0.03 | 0.03 | 0.03 |
|  |  | Thermoanaerobacteraceae | Caldanaerobacter | 0.04 | 0.05 | 0.03 | 0.02 | 0.03 | 0.03 | 0.06 | 0.08 | 0.03 | 0.04 | 0.03 | 0.02 | 0.04 | 0.04 | 0.04 | 0.04 |
|  |  |  | Carboxydothermus | 0.02 | 0.03 | 0.03 | 0.03 | 0.03 | 0.04 | 0.07 | 0.08 | 0.03 | 0.04 | 0.03 | 0.01 | 0.02 | 0.04 | 0.03 | 0.05 |
|  |  |  | Moorella | 0.02 | 0.03 | 0.02 | 0.06 | 0.03 | 0.04 | 0.06 | 0.10 | 0.02 | 0.04 | 0.01 | 0.01 | 0.02 | 0.02 | 0.02 | 0.04 |
|  |  |  | Thermoanaerobacter | 0.03 | 0.03 | 0.03 | 0.06 | 0.04 | 0.04 | 0.05 | 0.06 | 0.02 | 0.03 | 0.02 | 0.03 | 0.02 | 0.05 | 0.03 | 0.03 |
|  |  | Thermoanaerobacterales Family III. Incertae Sedis | Caldicellulosiruptor | 0.02 | 0.03 | 0.03 | 0.01 | 0.03 | 0.03 | 0.04 | 0.06 | 0.02 | 0.03 | 0.02 | 0.01 | 0.02 | 0.03 | 0.01 | 0.03 |
|  |  | Thermodesulfobiaceae | Coprothermobacter | 0.01 | 0.01 | 0.01 | 0.01 | 0.01 | 0.01 | 0.01 | 0.01 | 0.01 | 0.01 | 0.01 | 0.01 | 0.00 | 0.01 | 0.00 | 0.01 |
|  | Erysipelotrichi | Erysipelotrichaceae | Holdemania | 0.01 | 0.01 | 0.01 | 0.02 | 0.01 | 0.02 | 0.01 | 0.01 | 0.01 | 0.01 | 0.01 | 0.00 | 0.01 | 0.02 | 0.01 | 0.01 |
|  | Negativicutes | Veillonellaceae | Veillonella | 0.01 | 0.01 | 0.02 | 0.02 | 0.01 | 0.02 | 0.02 | 0.02 | 0.01 | 0.01 | 0.01 | 0.01 | 0.01 | 0.01 | 0.02 | 0.01 |
| Fusobacteria | Fusobacteriia | Fusobacteriaceae | Fusobacterium | 0.03 | 0.03 | 0.02 | 0.01 | 0.02 | 0.05 | 0.04 | 0.03 | 0.02 | 0.03 | 0.03 | 0.01 | 0.04 | 0.03 | 0.03 | 0.02 |
|  |  | Leptotrichiaceae | Leptotrichia | 0.01 | 0.01 | 0.01 | 0.00 | 0.02 | 0.01 | 0.01 | 0.02 | 0.01 | 0.01 | 0.01 | 0.01 | 0.01 | 0.01 | 0.01 | 0.01 |
|  |  |  | Sebaldella | 0.02 | 0.02 | 0.02 | 0.01 | 0.02 | 0.02 | 0.03 | 0.03 | 0.01 | 0.02 | 0.02 | 0.01 | 0.02 | 0.02 | 0.01 | 0.02 |
|  |  |  | Streptobacillus | 0.00 | 0.01 | 0.01 | 0.01 | 0.01 | 0.02 | 0.01 | 0.01 | 0.01 | 0.01 | 0.01 | 0.01 | 0.01 | 0.01 | 0.00 | 0.01 |
| Planctomycetes | Planctomycetia | Planctomycetaceae | Blastopirellula | 0.26 | 0.12 | 0.12 | 1.47 | 0.10 | 1.18 | 1.51 | 5.91 | 0.18 | 0.99 | 0.18 | 0.07 | 0.09 | 0.29 | 0.26 | 1.23 |
|  |  |  | Planctomyces | 0.12 | 0.06 | 0.06 | 0.44 | 0.06 | 0.41 | 0.65 | 2.06 | 0.07 | 0.37 | 0.09 | 0.05 | 0.06 | 0.12 | 0.10 | 0.50 |
|  |  |  | Rhodopirellula | 0.40 | 0.23 | 0.23 | 2.51 | 0.18 | 1.87 | 2.68 | 10.32 | 0.32 | 1.74 | 0.31 | 0.14 | 0.16 | 0.48 | 0.35 | 2.19 |
| Proteobacteria | Alphaproteobacteria | Acetobacteraceae | Acidiphilium | 0.18 | 0.17 | 0.10 | 0.09 | 0.20 | 0.14 | 0.19 | 0.15 | 0.10 | 0.14 | 0.15 | 0.13 | 0.22 | 0.14 | 0.07 | 0.18 |
|  |  |  | Gluconacetobacter | 0.00 | 0.00 | 0.00 | 0.00 | 0.00 | 0.00 | 0.00 | 0.00 | 0.00 | 0.00 | 0.00 | 0.00 | 0.00 | 0.00 | 0.00 | 0.00 |
|  |  |  | Gluconobacter | 0.07 | 0.07 | 0.04 | 0.02 | 0.08 | 0.06 | 0.07 | 0.06 | 0.04 | 0.05 | 0.06 | 0.05 | 0.08 | 0.06 | 0.03 | 0.07 |
|  |  |  | Granulibacter | 0.12 | 0.10 | 0.06 | 0.05 | 0.13 | 0.10 | 0.11 | 0.09 | 0.06 | 0.08 | 0.11 | 0.06 | 0.14 | 0.07 | 0.04 | 0.12 |
|  |  | Anaplasmataceae | Anaplasma | 0.01 | 0.02 | 0.01 | 0.01 | 0.01 | 0.01 | 0.01 | 0.01 | 0.01 | 0.01 | 0.02 | 0.01 | 0.02 | 0.02 | 0.00 | 0.01 |
|  |  |  | Ehrlichia | 0.03 | 0.04 | 0.03 | 0.00 | 0.04 | 0.02 | 0.02 | 0.02 | 0.02 | 0.02 | 0.03 | 0.02 | 0.03 | 0.04 | 0.01 | 0.02 |
|  |  |  | Neorickettsia | 0.01 | 0.01 | 0.01 | 0.00 | 0.01 | 0.00 | 0.01 | 0.01 | 0.01 | 0.01 | 0.01 | 0.01 | 0.01 | 0.01 | 0.00 | 0.00 |
|  |  |  | Wolbachia | 0.06 | 0.10 | 0.07 | 0.01 | 0.09 | 0.04 | 0.04 | 0.04 | 0.04 | 0.04 | 0.07 | 0.07 | 0.09 | 0.08 | 0.04 | 0.05 |
|  |  | Aurantimonadaceae | Aurantimonas | 0.22 | 0.18 | 0.12 | 0.17 | 0.19 | 0.24 | 0.23 | 0.28 | 0.12 | 0.19 | 0.23 | 0.12 | 0.23 | 0.18 | 0.07 | 0.23 |
|  |  | Bartonellaceae | Bartonella | 0.09 | 0.09 | 0.08 | 0.04 | 0.10 | 0.07 | 0.06 | 0.06 | 0.06 | 0.06 | 0.08 | 0.06 | 0.12 | 0.08 | 0.04 | 0.07 |
|  |  | Beijerinckiaceae | Beijerinckia | 0.00 | 0.00 | 0.00 | 0.00 | 0.00 | 0.00 | 0.00 | 0.00 | 0.00 | 0.00 | 0.00 | 0.00 | 0.00 | 0.00 | 0.00 | 0.00 |
|  |  |  | Methylocella | 0.08 | 0.09 | 0.05 | 0.11 | 0.08 | 0.09 | 0.10 | 0.10 | 0.05 | 0.07 | 0.09 | 0.04 | 0.10 | 0.08 | 0.04 | 0.09 |
|  |  | Bradyrhizobiaceae | Bradyrhizobium | 0.40 | 0.43 | 0.30 | 0.37 | 0.44 | 0.59 | 0.69 | 0.78 | 0.28 | 0.46 | 0.37 | 0.26 | 0.50 | 0.39 | 0.26 | 0.59 |
|  |  |  | Nitrobacter | 0.13 | 0.13 | 0.09 | 0.12 | 0.14 | 0.18 | 0.17 | 0.23 | 0.09 | 0.14 | 0.14 | 0.08 | 0.15 | 0.12 | 0.08 | 0.16 |
|  |  |  | Oligotropha | 0.08 | 0.07 | 0.05 | 0.08 | 0.08 | 0.08 | 0.09 | 0.09 | 0.05 | 0.06 | 0.06 | 0.05 | 0.10 | 0.07 | 0.04 | 0.08 |
|  |  |  | Rhodopseudomonas | 0.38 | 0.38 | 0.27 | 0.38 | 0.42 | 0.47 | 0.58 | 0.58 | 0.28 | 0.41 | 0.35 | 0.21 | 0.49 | 0.34 | 0.24 | 0.53 |
|  |  | Brucellaceae | Brucella | 0.31 | 0.30 | 0.21 | 0.24 | 0.28 | 0.32 | 0.33 | 0.47 | 0.19 | 0.27 | 0.25 | 0.16 | 0.38 | 0.22 | 0.13 | 0.38 |
|  |  |  | Ochrobactrum | 0.01 | 0.00 | 0.00 | 0.02 | 0.01 | 0.00 | 0.01 | 0.01 | 0.00 | 0.01 | 0.01 | 0.01 | 0.00 | 0.01 | 0.00 | 0.01 |
|  |  | Caulobacteraceae | Caulobacter | 0.44 | 0.34 | 0.21 | 0.26 | 0.29 | 0.32 | 0.40 | 0.35 | 0.20 | 0.32 | 0.32 | 0.19 | 0.45 | 0.29 | 0.16 | 0.38 |
|  |  |  | Phenylobacterium | 0.01 | 0.00 | 0.00 | 0.00 | 0.00 | 0.00 | 0.00 | 0.01 | 0.00 | 0.01 | 0.00 | 0.00 | 0.00 | 0.00 | 0.00 | 0.01 |
|  |  | Erythrobacteraceae | Erythrobacter | 0.51 | 0.31 | 0.28 | 0.59 | 0.31 | 0.55 | 0.57 | 0.50 | 0.25 | 0.57 | 0.49 | 0.23 | 0.43 | 0.31 | 0.34 | 0.52 |
|  |  | Hyphomonadaceae | Hyphomonas | 0.32 | 0.21 | 0.15 | 0.29 | 0.31 | 0.36 | 0.56 | 0.36 | 0.18 | 0.37 | 0.39 | 0.19 | 0.39 | 0.19 | 0.19 | 0.83 |
|  |  |  | Maricaulis | 0.22 | 0.18 | 0.14 | 0.14 | 0.21 | 0.26 | 0.33 | 0.30 | 0.14 | 0.23 | 0.16 | 0.17 | 0.24 | 0.18 | 0.09 | 0.29 |
|  |  |  | Oceanicaulis | 0.16 | 0.13 | 0.08 | 0.08 | 0.15 | 0.17 | 0.19 | 0.17 | 0.09 | 0.14 | 0.12 | 0.09 | 0.17 | 0.12 | 0.09 | 0.17 |
|  |  | Methylobacteriaceae | Methylobacterium | 0.23 | 0.19 | 0.15 | 0.33 | 0.21 | 0.31 | 0.37 | 0.43 | 0.15 | 0.24 | 0.25 | 0.15 | 0.29 | 0.23 | 0.12 | 0.31 |
|  |  | Parvularculaceae | Parvularcula | 0.12 | 0.10 | 0.08 | 0.07 | 0.13 | 0.18 | 0.15 | 0.14 | 0.07 | 0.13 | 0.12 | 0.05 | 0.15 | 0.11 | 0.05 | 0.14 |
|  |  | Phyllobacteriaceae | Chelativorans | 0.21 | 0.19 | 0.14 | 0.21 | 0.20 | 0.24 | 0.26 | 0.34 | 0.13 | 0.21 | 0.19 | 0.10 | 0.23 | 0.17 | 0.10 | 0.27 |
|  |  |  | Mesorhizobium | 0.32 | 0.32 | 0.22 | 0.29 | 0.35 | 0.38 | 0.46 | 0.59 | 0.22 | 0.38 | 0.32 | 0.17 | 0.36 | 0.28 | 0.14 | 0.46 |
|  |  |  | Parvibaculum | 0.79 | 0.65 | 0.43 | 0.26 | 0.80 | 0.54 | 0.77 | 0.57 | 0.51 | 0.56 | 0.63 | 0.36 | 1.11 | 0.59 | 0.21 | 0.74 |
|  |  | Rhizobiaceae | Agrobacterium | 0.27 | 0.25 | 0.18 | 0.26 | 0.27 | 0.23 | 0.22 | 0.28 | 0.15 | 0.19 | 0.28 | 0.16 | 0.29 | 0.24 | 0.11 | 0.27 |
|  |  |  | Rhizobium | 0.26 | 0.22 | 0.18 | 0.30 | 0.24 | 0.34 | 0.32 | 0.46 | 0.16 | 0.26 | 0.24 | 0.14 | 0.26 | 0.22 | 0.12 | 0.31 |
|  |  |  | Sinorhizobium | 0.36 | 0.33 | 0.23 | 0.32 | 0.34 | 0.43 | 0.43 | 0.63 | 0.23 | 0.34 | 0.28 | 0.15 | 0.39 | 0.24 | 0.15 | 0.45 |
|  |  | Rhodobacteraceae | Dinoroseobacter | 0.47 | 0.55 | 0.52 | 0.43 | 0.61 | 0.98 | 0.77 | 0.81 | 0.42 | 0.67 | 0.56 | 0.28 | 0.57 | 0.55 | 0.33 | 0.85 |
|  |  |  | Loktanella | 0.24 | 0.27 | 0.24 | 0.27 | 0.34 | 0.59 | 0.34 | 0.29 | 0.22 | 0.29 | 0.29 | 0.18 | 0.30 | 0.35 | 0.23 | 0.35 |
|  |  |  | Maritimibacter | 0.29 | 0.28 | 0.27 | 0.39 | 0.35 | 0.64 | 0.41 | 0.42 | 0.24 | 0.37 | 0.33 | 0.20 | 0.31 | 0.31 | 0.27 | 0.44 |
|  |  |  | Oceanicola | 0.27 | 0.26 | 0.29 | 0.32 | 0.31 | 0.57 | 0.38 | 0.37 | 0.21 | 0.36 | 0.31 | 0.23 | 0.28 | 0.30 | 0.30 | 0.38 |
|  |  |  | Paracoccus | 0.23 | 0.17 | 0.15 | 0.21 | 0.20 | 0.29 | 0.25 | 0.28 | 0.12 | 0.21 | 0.18 | 0.15 | 0.21 | 0.17 | 0.15 | 0.28 |
|  |  |  | Rhodobacter | 0.39 | 0.42 | 0.33 | 0.41 | 0.41 | 0.66 | 0.54 | 0.57 | 0.31 | 0.46 | 0.38 | 0.23 | 0.47 | 0.37 | 0.27 | 0.63 |
|  |  |  | Roseobacter | 1.22 | 1.22 | 1.26 | 1.40 | 1.20 | 2.70 | 1.69 | 1.84 | 0.89 | 1.90 | 1.29 | 0.69 | 1.32 | 1.17 | 1.30 | 1.84 |
|  |  |  | Roseovarius | 0.71 | 0.74 | 0.76 | 0.97 | 0.88 | 1.75 | 1.16 | 1.15 | 0.59 | 1.06 | 0.89 | 0.51 | 0.79 | 0.81 | 0.71 | 1.13 |
|  |  |  | Ruegeria | 1.49 | 1.60 | 1.84 | 1.31 | 1.59 | 4.27 | 2.47 | 2.57 | 1.27 | 2.74 | 1.76 | 2.08 | 1.70 | 1.52 | 1.36 | 2.83 |
|  |  |  | Sulfitobacter | 0.31 | 0.25 | 0.26 | 0.55 | 0.27 | 0.68 | 0.40 | 0.39 | 0.22 | 0.63 | 0.48 | 0.26 | 0.31 | 1.08 | 1.70 | 0.42 |
|  |  | Rhodospirillaceae | Magnetospirillum | 0.75 | 0.61 | 0.37 | 0.31 | 0.76 | 0.65 | 0.85 | 0.68 | 0.41 | 0.57 | 0.64 | 0.41 | 0.84 | 0.59 | 0.30 | 0.74 |
|  |  |  | Rhodospirillum | 0.76 | 0.65 | 0.38 | 0.35 | 0.86 | 0.62 | 0.88 | 0.66 | 0.42 | 0.61 | 0.72 | 0.39 | 0.90 | 0.58 | 0.26 | 0.79 |
|  |  | Rickettsiaceae | Orientia | 0.01 | 0.02 | 0.02 | 0.02 | 0.01 | 0.02 | 0.01 | 0.01 | 0.01 | 0.01 | 0.02 | 0.01 | 0.01 | 0.02 | 0.00 | 0.01 |
|  |  |  | Rickettsia | 0.12 | 0.20 | 0.12 | 0.04 | 0.17 | 0.09 | 0.09 | 0.07 | 0.09 | 0.08 | 0.13 | 0.08 | 0.16 | 0.13 | 0.05 | 0.10 |
|  |  | Sphingomonadaceae | Novosphingobium | 0.16 | 0.11 | 0.09 | 0.18 | 0.12 | 0.18 | 0.22 | 0.16 | 0.09 | 0.16 | 0.16 | 0.08 | 0.14 | 0.12 | 0.11 | 0.17 |
|  |  |  | Sphingomonas | 0.19 | 0.15 | 0.10 | 0.17 | 0.14 | 0.19 | 0.27 | 0.22 | 0.10 | 0.20 | 0.20 | 0.10 | 0.19 | 0.15 | 0.11 | 0.21 |
|  |  |  | Sphingopyxis | 0.15 | 0.11 | 0.08 | 0.12 | 0.11 | 0.15 | 0.18 | 0.16 | 0.09 | 0.15 | 0.13 | 0.08 | 0.16 | 0.10 | 0.08 | 0.16 |
|  |  |  | Zymomonas | 0.06 | 0.08 | 0.04 | 0.02 | 0.05 | 0.05 | 0.04 | 0.04 | 0.04 | 0.04 | 0.06 | 0.06 | 0.10 | 0.05 | 0.02 | 0.05 |
|  |  | unclassified (derived from Alphaproteobacteria) | Candidatus Pelagibacter | 18.85 | 27.15 | 22.36 | 0.54 | 26.17 | 3.32 | 3.25 | 0.44 | 12.16 | 6.62 | 17.51 | 12.77 | 24.31 | 17.74 | 5.72 | 9.28 |
|  |  | Xanthobacteraceae | Azorhizobium | 0.16 | 0.12 | 0.09 | 0.15 | 0.19 | 0.17 | 0.20 | 0.20 | 0.10 | 0.14 | 0.17 | 0.12 | 0.18 | 0.15 | 0.09 | 0.16 |
|  |  |  | Xanthobacter | 0.00 | 0.00 | 0.00 | 0.00 | 0.00 | 0.00 | 0.01 | 0.01 | 0.00 | 0.00 | 0.00 | 0.00 | 0.00 | 0.00 | 0.00 | 0.00 |
|  | Betaproteobacteria | Alcaligenaceae | Achromobacter | 0.00 | 0.00 | 0.00 | 0.00 | 0.00 | 0.00 | 0.00 | 0.00 | 0.00 | 0.00 | 0.00 | 0.00 | 0.00 | 0.00 | 0.00 | 0.00 |
|  |  |  | Bordetella | 0.18 | 0.18 | 0.13 | 0.14 | 0.20 | 0.21 | 0.29 | 0.29 | 0.10 | 0.17 | 0.16 | 0.13 | 0.18 | 0.15 | 0.11 | 0.23 |
|  |  | Burkholderiaceae | Burkholderia | 0.35 | 0.28 | 0.23 | 0.35 | 0.35 | 0.61 | 0.69 | 0.65 | 0.25 | 0.45 | 0.37 | 0.29 | 0.32 | 0.38 | 0.31 | 0.46 |
|  |  |  | Cupriavidus | 0.17 | 0.15 | 0.10 | 0.14 | 0.19 | 0.22 | 0.31 | 0.26 | 0.11 | 0.20 | 0.14 | 0.10 | 0.17 | 0.16 | 0.10 | 0.22 |
|  |  |  | Polynucleobacter | 0.03 | 0.05 | 0.04 | 0.04 | 0.06 | 0.04 | 0.05 | 0.05 | 0.03 | 0.03 | 0.04 | 0.03 | 0.04 | 0.04 | 0.02 | 0.05 |
|  |  |  | Ralstonia | 0.07 | 0.05 | 0.04 | 0.09 | 0.05 | 0.07 | 0.10 | 0.10 | 0.04 | 0.07 | 0.05 | 0.05 | 0.05 | 0.06 | 0.04 | 0.07 |
|  |  | Comamonadaceae | Acidovorax | 0.08 | 0.05 | 0.05 | 0.10 | 0.07 | 0.12 | 0.13 | 0.13 | 0.05 | 0.09 | 0.08 | 0.03 | 0.07 | 0.09 | 0.06 | 0.10 |
|  |  |  | Albidiferax | 0.06 | 0.05 | 0.04 | 0.06 | 0.05 | 0.08 | 0.12 | 0.12 | 0.04 | 0.08 | 0.05 | 0.04 | 0.06 | 0.06 | 0.05 | 0.09 |
|  |  |  | Comamonas | 0.00 | 0.00 | 0.00 | 0.00 | 0.00 | 0.00 | 0.00 | 0.00 | 0.00 | 0.00 | 0.00 | 0.00 | 0.00 | 0.00 | 0.00 | 0.00 |
|  |  |  | Delftia | 0.05 | 0.04 | 0.03 | 0.07 | 0.04 | 0.07 | 0.09 | 0.08 | 0.04 | 0.06 | 0.06 | 0.04 | 0.05 | 0.06 | 0.04 | 0.06 |
|  |  |  | Polaromonas | 0.09 | 0.08 | 0.06 | 0.11 | 0.08 | 0.12 | 0.16 | 0.16 | 0.06 | 0.10 | 0.07 | 0.04 | 0.08 | 0.09 | 0.07 | 0.12 |
|  |  |  | Verminephrobacter | 0.06 | 0.06 | 0.04 | 0.06 | 0.07 | 0.09 | 0.10 | 0.09 | 0.05 | 0.07 | 0.07 | 0.03 | 0.07 | 0.05 | 0.04 | 0.09 |
|  |  | Hydrogenophilaceae | Thiobacillus | 0.06 | 0.06 | 0.04 | 0.07 | 0.06 | 0.13 | 0.20 | 0.18 | 0.05 | 0.11 | 0.06 | 0.04 | 0.06 | 0.06 | 0.05 | 0.10 |
|  |  | Methylophilaceae | Methylobacillus | 0.08 | 0.06 | 0.06 | 0.05 | 0.06 | 0.10 | 0.11 | 0.09 | 0.05 | 0.08 | 0.07 | 0.07 | 0.07 | 0.07 | 0.06 | 0.08 |
|  |  | Neisseriaceae | Chromobacterium | 0.08 | 0.06 | 0.05 | 0.09 | 0.07 | 0.08 | 0.15 | 0.13 | 0.07 | 0.08 | 0.07 | 0.04 | 0.07 | 0.07 | 0.06 | 0.10 |
|  |  |  | Laribacter | 0.00 | 0.00 | 0.00 | 0.00 | 0.00 | 0.00 | 0.00 | 0.00 | 0.00 | 0.00 | 0.00 | 0.00 | 0.00 | 0.00 | 0.00 | 0.00 |
|  |  |  | Neisseria | 0.03 | 0.04 | 0.04 | 0.02 | 0.05 | 0.04 | 0.06 | 0.04 | 0.03 | 0.04 | 0.04 | 0.04 | 0.04 | 0.06 | 0.05 | 0.04 |
|  |  | Nitrosomonadaceae | Nitrosomonas | 0.09 | 0.07 | 0.07 | 0.08 | 0.08 | 0.12 | 0.16 | 0.17 | 0.06 | 0.10 | 0.08 | 0.05 | 0.09 | 0.09 | 0.06 | 0.11 |
|  |  |  | Nitrosospira | 0.06 | 0.04 | 0.03 | 0.06 | 0.04 | 0.09 | 0.13 | 0.12 | 0.05 | 0.07 | 0.04 | 0.02 | 0.04 | 0.05 | 0.05 | 0.08 |
|  |  | Oxalobacteraceae | Collimonas | 0.00 | 0.00 | 0.00 | 0.00 | 0.00 | 0.00 | 0.00 | 0.00 | 0.00 | 0.00 | 0.00 | 0.00 | 0.00 | 0.00 | 0.00 | 0.00 |
|  |  |  | Herminiimonas | 0.05 | 0.04 | 0.03 | 0.06 | 0.04 | 0.05 | 0.07 | 0.06 | 0.03 | 0.05 | 0.03 | 0.03 | 0.04 | 0.04 | 0.04 | 0.05 |
|  |  |  | Janthinobacterium | 0.05 | 0.04 | 0.03 | 0.05 | 0.04 | 0.07 | 0.08 | 0.08 | 0.04 | 0.06 | 0.05 | 0.04 | 0.04 | 0.05 | 0.03 | 0.05 |
|  |  | Rhodocyclaceae | Aromatoleum | 0.09 | 0.07 | 0.08 | 0.10 | 0.08 | 0.12 | 0.17 | 0.16 | 0.05 | 0.11 | 0.09 | 0.04 | 0.08 | 0.07 | 0.07 | 0.11 |
|  |  |  | Azoarcus | 0.09 | 0.06 | 0.06 | 0.11 | 0.07 | 0.13 | 0.19 | 0.17 | 0.06 | 0.11 | 0.07 | 0.07 | 0.07 | 0.10 | 0.05 | 0.12 |
|  |  |  | Dechloromonas | 0.07 | 0.06 | 0.04 | 0.07 | 0.07 | 0.15 | 0.18 | 0.16 | 0.06 | 0.11 | 0.05 | 0.04 | 0.06 | 0.06 | 0.05 | 0.11 |
|  |  | unclassified (derived from Burkholderiales) | Methylibium | 0.06 | 0.04 | 0.04 | 0.08 | 0.05 | 0.08 | 0.12 | 0.09 | 0.04 | 0.07 | 0.04 | 0.06 | 0.05 | 0.05 | 0.03 | 0.07 |
|  | Deltaproteobacteria | Bacteriovoracaceae | Bacteriovorax | 0.10 | 0.08 | 0.07 | 0.08 | 0.10 | 0.19 | 0.36 | 0.26 | 0.08 | 0.18 | 0.12 | 0.07 | 0.11 | 0.13 | 0.07 | 0.18 |
|  |  | Bdellovibrionaceae | Bdellovibrio | 0.07 | 0.07 | 0.05 | 0.07 | 0.07 | 0.10 | 0.22 | 0.20 | 0.05 | 0.11 | 0.07 | 0.06 | 0.09 | 0.08 | 0.07 | 0.12 |
|  |  | Desulfobacteraceae | Desulfatibacillum | 0.08 | 0.08 | 0.06 | 0.14 | 0.07 | 0.19 | 0.27 | 0.33 | 0.08 | 0.18 | 0.10 | 0.06 | 0.07 | 0.12 | 0.09 | 0.14 |
|  |  |  | Desulfobacterium | 0.00 | 0.00 | 0.00 | 0.00 | 0.00 | 0.00 | 0.00 | 0.00 | 0.00 | 0.00 | 0.00 | 0.00 | 0.00 | 0.00 | 0.00 | 0.00 |
|  |  |  | Desulfococcus | 0.06 | 0.05 | 0.04 | 0.09 | 0.06 | 0.11 | 0.20 | 0.27 | 0.04 | 0.13 | 0.05 | 0.03 | 0.05 | 0.07 | 0.05 | 0.10 |
|  |  | Desulfobulbaceae | Desulfotalea | 0.08 | 0.07 | 0.05 | 0.17 | 0.07 | 0.25 | 0.27 | 0.54 | 0.09 | 0.20 | 0.08 | 0.04 | 0.09 | 0.11 | 0.07 | 0.14 |
|  |  | Desulfohalobiaceae | Desulfohalobium | 0.03 | 0.02 | 0.02 | 0.05 | 0.02 | 0.06 | 0.06 | 0.09 | 0.02 | 0.04 | 0.02 | 0.02 | 0.02 | 0.04 | 0.03 | 0.04 |
|  |  | Desulfomicrobiaceae | Desulfomicrobium | 0.03 | 0.02 | 0.02 | 0.03 | 0.03 | 0.06 | 0.09 | 0.13 | 0.02 | 0.06 | 0.03 | 0.02 | 0.03 | 0.05 | 0.03 | 0.05 |
|  |  | Desulfovibrionaceae | Desulfovibrio | 0.08 | 0.07 | 0.07 | 0.13 | 0.08 | 0.18 | 0.22 | 0.33 | 0.07 | 0.14 | 0.09 | 0.05 | 0.08 | 0.10 | 0.08 | 0.14 |
|  |  |  | Lawsonia | 0.01 | 0.01 | 0.01 | 0.01 | 0.02 | 0.01 | 0.02 | 0.02 | 0.01 | 0.01 | 0.01 | 0.01 | 0.01 | 0.01 | 0.01 | 0.01 |
|  |  | Desulfuromonadaceae | Desulfuromonas | 0.09 | 0.08 | 0.06 | 0.10 | 0.07 | 0.19 | 0.26 | 0.30 | 0.06 | 0.15 | 0.11 | 0.06 | 0.07 | 0.09 | 0.07 | 0.14 |
|  |  | Geobacteraceae | Geobacter | 0.15 | 0.16 | 0.12 | 0.26 | 0.14 | 0.36 | 0.57 | 0.71 | 0.14 | 0.33 | 0.15 | 0.13 | 0.14 | 0.19 | 0.18 | 0.33 |
|  |  | Kofleriaceae | Haliangium | 0.12 | 0.10 | 0.08 | 0.19 | 0.11 | 0.34 | 0.57 | 0.57 | 0.10 | 0.27 | 0.12 | 0.09 | 0.09 | 0.14 | 0.10 | 0.31 |
|  |  | Myxococcaceae | Anaeromyxobacter | 0.10 | 0.07 | 0.07 | 0.18 | 0.09 | 0.26 | 0.45 | 0.57 | 0.09 | 0.21 | 0.09 | 0.05 | 0.08 | 0.14 | 0.09 | 0.27 |
|  |  |  | Myxococcus | 0.08 | 0.07 | 0.06 | 0.15 | 0.07 | 0.22 | 0.41 | 0.45 | 0.07 | 0.19 | 0.07 | 0.07 | 0.07 | 0.10 | 0.10 | 0.24 |
|  |  | Pelobacteraceae | Pelobacter | 0.08 | 0.08 | 0.06 | 0.13 | 0.09 | 0.18 | 0.33 | 0.40 | 0.08 | 0.17 | 0.07 | 0.04 | 0.08 | 0.12 | 0.06 | 0.19 |
|  |  | Polyangiaceae | Sorangium | 0.10 | 0.06 | 0.05 | 0.20 | 0.08 | 0.26 | 0.49 | 0.50 | 0.09 | 0.25 | 0.09 | 0.05 | 0.06 | 0.10 | 0.08 | 0.27 |
|  |  | Syntrophaceae | Syntrophus | 0.03 | 0.03 | 0.03 | 0.06 | 0.03 | 0.07 | 0.12 | 0.19 | 0.03 | 0.07 | 0.02 | 0.02 | 0.03 | 0.04 | 0.04 | 0.07 |
|  |  | Syntrophobacteraceae | Syntrophobacter | 0.04 | 0.03 | 0.03 | 0.06 | 0.03 | 0.12 | 0.16 | 0.27 | 0.04 | 0.10 | 0.05 | 0.02 | 0.03 | 0.06 | 0.04 | 0.09 |
|  | Epsilonproteobacteria | Campylobacteraceae | Arcobacter | 0.04 | 0.04 | 0.03 | 0.02 | 0.03 | 0.04 | 0.03 | 0.06 | 0.04 | 0.04 | 0.03 | 0.01 | 0.04 | 0.04 | 0.02 | 0.02 |
|  |  |  | Campylobacter | 0.07 | 0.07 | 0.06 | 0.05 | 0.08 | 0.07 | 0.07 | 0.07 | 0.04 | 0.05 | 0.07 | 0.05 | 0.06 | 0.08 | 0.07 | 0.05 |
|  |  |  | Sulfurospirillum | 0.02 | 0.03 | 0.02 | 0.02 | 0.02 | 0.02 | 0.02 | 0.02 | 0.02 | 0.02 | 0.02 | 0.01 | 0.02 | 0.02 | 0.02 | 0.02 |
|  |  | Helicobacteraceae | Helicobacter | 0.02 | 0.03 | 0.03 | 0.01 | 0.03 | 0.02 | 0.03 | 0.02 | 0.02 | 0.02 | 0.02 | 0.02 | 0.02 | 0.03 | 0.02 | 0.02 |
|  |  |  | Sulfurimonas | 0.02 | 0.02 | 0.02 | 0.02 | 0.02 | 0.02 | 0.03 | 0.02 | 0.02 | 0.02 | 0.01 | 0.02 | 0.02 | 0.02 | 0.02 | 0.02 |
|  |  |  | Wolinella | 0.02 | 0.02 | 0.01 | 0.02 | 0.01 | 0.02 | 0.03 | 0.02 | 0.01 | 0.02 | 0.01 | 0.01 | 0.02 | 0.02 | 0.02 | 0.02 |
|  |  | unclassified (derived from Epsilonproteobacteria) | Nitratiruptor | 0.02 | 0.03 | 0.02 | 0.00 | 0.03 | 0.02 | 0.04 | 0.04 | 0.02 | 0.02 | 0.02 | 0.01 | 0.02 | 0.02 | 0.03 | 0.02 |
|  |  |  | Sulfurovum | 0.03 | 0.03 | 0.03 | 0.04 | 0.02 | 0.04 | 0.07 | 0.07 | 0.03 | 0.05 | 0.03 | 0.01 | 0.03 | 0.03 | 0.04 | 0.04 |
|  | Gammaproteobacteria | Acidithiobacillaceae | Acidithiobacillus | 0.03 | 0.02 | 0.02 | 0.03 | 0.03 | 0.05 | 0.07 | 0.07 | 0.02 | 0.04 | 0.03 | 0.02 | 0.02 | 0.05 | 0.02 | 0.04 |
|  |  | Aeromonadaceae | Aeromonas | 0.16 | 0.14 | 0.13 | 0.09 | 0.13 | 0.19 | 0.26 | 0.18 | 0.19 | 0.18 | 0.17 | 0.11 | 0.16 | 0.13 | 0.06 | 0.21 |
|  |  | Alcanivoracaceae | Alcanivorax | 0.87 | 0.25 | 0.20 | 0.30 | 0.51 | 0.33 | 0.66 | 0.27 | 0.21 | 0.62 | 0.48 | 0.48 | 0.54 | 0.26 | 0.34 | 0.81 |
|  |  |  | Kangiella | 0.14 | 0.16 | 0.15 | 0.12 | 0.17 | 0.27 | 0.32 | 0.23 | 0.13 | 0.21 | 0.18 | 0.13 | 0.15 | 0.17 | 0.09 | 0.22 |
|  |  | Alteromonadaceae | Alteromonas | 15.70 | 8.39 | 15.87 | 2.41 | 12.37 | 2.10 | 3.24 | 0.31 | 4.07 | 9.89 | 18.26 | 36.24 | 12.14 | 10.15 | 5.79 | 10.61 |
|  |  |  | Marinobacter | 0.89 | 0.31 | 0.33 | 0.26 | 0.45 | 0.44 | 0.69 | 0.37 | 0.25 | 0.61 | 0.55 | 0.45 | 0.67 | 0.49 | 0.29 | 0.51 |
|  |  |  | Saccharophagus | 0.64 | 0.52 | 0.47 | 0.33 | 0.51 | 0.76 | 1.38 | 0.56 | 0.48 | 0.77 | 0.57 | 0.50 | 0.55 | 0.51 | 0.36 | 0.82 |
|  |  | Cardiobacteriaceae | Dichelobacter | 0.02 | 0.02 | 0.02 | 0.02 | 0.03 | 0.02 | 0.04 | 0.03 | 0.02 | 0.03 | 0.02 | 0.01 | 0.02 | 0.03 | 0.01 | 0.03 |
|  |  | Chromatiaceae | Nitrosococcus | 0.15 | 0.13 | 0.09 | 0.19 | 0.14 | 0.24 | 0.51 | 0.45 | 0.11 | 0.26 | 0.10 | 0.06 | 0.12 | 0.17 | 0.08 | 0.28 |
|  |  | Colwelliaceae | Colwellia | 0.54 | 0.49 | 0.57 | 0.13 | 0.39 | 0.66 | 0.80 | 0.50 | 0.95 | 0.82 | 0.69 | 0.54 | 0.45 | 0.59 | 0.20 | 0.63 |
|  |  | Coxiellaceae | Coxiella | 0.06 | 0.08 | 0.06 | 0.18 | 0.06 | 0.08 | 0.17 | 0.46 | 0.07 | 0.11 | 0.06 | 0.04 | 0.06 | 0.09 | 0.10 | 0.11 |
|  |  | Ectothiorhodospiraceae | Alkalilimnicola | 0.17 | 0.12 | 0.09 | 0.14 | 0.10 | 0.27 | 0.48 | 0.36 | 0.11 | 0.25 | 0.15 | 0.09 | 0.11 | 0.15 | 0.13 | 0.27 |
|  |  |  | Halorhodospira | 0.09 | 0.09 | 0.05 | 0.07 | 0.08 | 0.14 | 0.26 | 0.23 | 0.07 | 0.14 | 0.07 | 0.05 | 0.07 | 0.10 | 0.06 | 0.15 |
|  |  |  | Nitrococcus | 0.09 | 0.06 | 0.05 | 0.11 | 0.08 | 0.21 | 0.28 | 0.28 | 0.07 | 0.15 | 0.10 | 0.05 | 0.06 | 0.09 | 0.10 | 0.16 |
|  |  |  | Thioalkalivibrio | 0.19 | 0.14 | 0.11 | 0.23 | 0.14 | 0.38 | 0.69 | 0.51 | 0.16 | 0.35 | 0.14 | 0.08 | 0.13 | 0.18 | 0.15 | 0.36 |
|  |  | Enterobacteriaceae | Buchnera | 0.02 | 0.04 | 0.03 | 0.01 | 0.03 | 0.01 | 0.02 | 0.01 | 0.02 | 0.01 | 0.02 | 0.02 | 0.03 | 0.03 | 0.01 | 0.02 |
|  |  |  | Candidatus Blochmannia | 0.00 | 0.00 | 0.00 | 0.00 | 0.01 | 0.00 | 0.00 | 0.00 | 0.00 | 0.00 | 0.00 | 0.00 | 0.00 | 0.00 | 0.00 | 0.00 |
|  |  |  | Citrobacter | 0.02 | 0.02 | 0.01 | 0.04 | 0.02 | 0.03 | 0.03 | 0.02 | 0.02 | 0.02 | 0.02 | 0.01 | 0.02 | 0.03 | 0.01 | 0.02 |
|  |  |  | Enterobacter | 0.02 | 0.02 | 0.02 | 0.02 | 0.03 | 0.04 | 0.04 | 0.03 | 0.03 | 0.02 | 0.02 | 0.02 | 0.02 | 0.03 | 0.02 | 0.03 |
|  |  |  | Erwinia | 0.00 | 0.00 | 0.00 | 0.00 | 0.00 | 0.00 | 0.00 | 0.00 | 0.00 | 0.00 | 0.00 | 0.00 | 0.00 | 0.00 | 0.00 | 0.00 |
|  |  |  | Escherichia | 0.08 | 0.06 | 0.06 | 0.06 | 0.05 | 0.07 | 0.09 | 0.08 | 0.08 | 0.08 | 0.08 | 0.05 | 0.07 | 0.07 | 0.06 | 0.07 |
|  |  |  | Klebsiella | 0.05 | 0.03 | 0.02 | 0.05 | 0.03 | 0.05 | 0.05 | 0.05 | 0.03 | 0.04 | 0.04 | 0.03 | 0.03 | 0.03 | 0.03 | 0.04 |
|  |  |  | Pantoea | 0.00 | 0.00 | 0.00 | 0.00 | 0.00 | 0.00 | 0.00 | 0.00 | 0.00 | 0.00 | 0.00 | 0.00 | 0.00 | 0.00 | 0.00 | 0.00 |
|  |  |  | Pectobacterium | 0.04 | 0.03 | 0.03 | 0.03 | 0.03 | 0.07 | 0.07 | 0.06 | 0.04 | 0.05 | 0.04 | 0.04 | 0.04 | 0.04 | 0.03 | 0.04 |
|  |  |  | Photorhabdus | 0.06 | 0.06 | 0.05 | 0.04 | 0.06 | 0.06 | 0.10 | 0.07 | 0.06 | 0.07 | 0.07 | 0.06 | 0.06 | 0.06 | 0.03 | 0.06 |
|  |  |  | Proteus | 0.04 | 0.03 | 0.04 | 0.04 | 0.04 | 0.04 | 0.05 | 0.04 | 0.05 | 0.05 | 0.05 | 0.03 | 0.05 | 0.03 | 0.04 | 0.04 |
|  |  |  | Salmonella | 0.10 | 0.09 | 0.09 | 0.06 | 0.07 | 0.10 | 0.11 | 0.07 | 0.14 | 0.09 | 0.11 | 0.09 | 0.10 | 0.08 | 0.09 | 0.09 |
|  |  |  | Serratia | 0.06 | 0.04 | 0.04 | 0.04 | 0.05 | 0.10 | 0.10 | 0.08 | 0.06 | 0.07 | 0.07 | 0.05 | 0.05 | 0.07 | 0.04 | 0.07 |
|  |  |  | Shigella | 0.06 | 0.03 | 0.02 | 0.04 | 0.04 | 0.04 | 0.06 | 0.05 | 0.04 | 0.04 | 0.06 | 0.01 | 0.05 | 0.04 | 0.04 | 0.05 |
|  |  |  | Sodalis | 0.02 | 0.01 | 0.01 | 0.02 | 0.01 | 0.02 | 0.03 | 0.02 | 0.01 | 0.02 | 0.02 | 0.02 | 0.01 | 0.02 | 0.01 | 0.02 |
|  |  |  | Wigglesworthia | 0.00 | 0.01 | 0.01 | 0.00 | 0.01 | 0.00 | 0.00 | 0.00 | 0.00 | 0.00 | 0.00 | 0.01 | 0.00 | 0.00 | 0.00 | 0.00 |
|  |  |  | Yersinia | 0.13 | 0.10 | 0.10 | 0.10 | 0.10 | 0.18 | 0.16 | 0.14 | 0.13 | 0.13 | 0.14 | 0.12 | 0.12 | 0.14 | 0.08 | 0.13 |
|  |  | Francisellaceae | Francisella | 0.09 | 0.14 | 0.11 | 0.04 | 0.16 | 0.08 | 0.12 | 0.10 | 0.09 | 0.08 | 0.09 | 0.08 | 0.11 | 0.11 | 0.08 | 0.09 |
|  |  | Hahellaceae | Hahella | 0.41 | 0.37 | 0.38 | 0.20 | 0.35 | 0.52 | 0.80 | 0.51 | 0.34 | 0.49 | 0.34 | 0.24 | 0.35 | 0.40 | 0.20 | 0.52 |
|  |  | Halomonadaceae | Chromohalobacter | 1.11 | 0.26 | 0.24 | 0.27 | 0.27 | 0.28 | 0.39 | 0.27 | 0.20 | 0.48 | 0.70 | 0.21 | 0.27 | 0.24 | 0.61 | 0.37 |
|  |  | Idiomarinaceae | Idiomarina | 0.51 | 0.38 | 0.36 | 0.13 | 0.32 | 0.31 | 0.40 | 0.25 | 0.32 | 0.44 | 0.45 | 0.34 | 0.45 | 0.27 | 0.17 | 0.45 |
|  |  | Legionellaceae | Fluoribacter | 0.00 | 0.00 | 0.00 | 0.00 | 0.00 | 0.00 | 0.00 | 0.00 | 0.00 | 0.00 | 0.00 | 0.00 | 0.00 | 0.00 | 0.00 | 0.00 |
|  |  |  | Legionella | 0.11 | 0.14 | 0.11 | 0.13 | 0.13 | 0.19 | 0.23 | 0.42 | 0.09 | 0.19 | 0.12 | 0.09 | 0.11 | 0.13 | 0.10 | 0.15 |
|  |  | Methylococcaceae | Methylococcus | 0.14 | 0.13 | 0.11 | 0.11 | 0.14 | 0.23 | 0.43 | 0.37 | 0.12 | 0.24 | 0.15 | 0.11 | 0.12 | 0.13 | 0.09 | 0.25 |
|  |  | Moraxellaceae | Acinetobacter | 0.20 | 0.11 | 0.09 | 0.26 | 0.10 | 0.15 | 0.15 | 0.13 | 0.09 | 0.11 | 0.11 | 0.11 | 0.11 | 0.11 | 0.13 | 0.11 |
|  |  |  | Moraxella | 0.00 | 0.00 | 0.00 | 0.00 | 0.00 | 0.00 | 0.00 | 0.00 | 0.00 | 0.00 | 0.00 | 0.00 | 0.00 | 0.00 | 0.00 | 0.00 |
|  |  |  | Psychrobacter | 1.00 | 0.19 | 0.17 | 1.81 | 0.18 | 0.17 | 0.17 | 0.30 | 0.21 | 0.17 | 0.19 | 0.13 | 0.33 | 0.16 | 0.30 | 0.14 |
|  |  | Pasteurellaceae | Actinobacillus | 0.04 | 0.05 | 0.03 | 0.03 | 0.04 | 0.05 | 0.06 | 0.05 | 0.06 | 0.04 | 0.04 | 0.04 | 0.04 | 0.04 | 0.04 | 0.05 |
|  |  |  | Aggregatibacter | 0.00 | 0.00 | 0.00 | 0.00 | 0.01 | 0.00 | 0.01 | 0.01 | 0.00 | 0.01 | 0.01 | 0.01 | 0.00 | 0.01 | 0.00 | 0.00 |
|  |  |  | Basfia | 0.02 | 0.02 | 0.01 | 0.02 | 0.02 | 0.02 | 0.03 | 0.02 | 0.02 | 0.02 | 0.01 | 0.01 | 0.02 | 0.03 | 0.02 | 0.02 |
|  |  |  | Haemophilus | 0.09 | 0.08 | 0.06 | 0.06 | 0.08 | 0.10 | 0.09 | 0.08 | 0.06 | 0.07 | 0.07 | 0.08 | 0.07 | 0.06 | 0.08 | 0.07 |
|  |  |  | Histophilus | 0.01 | 0.01 | 0.01 | 0.00 | 0.01 | 0.02 | 0.01 | 0.01 | 0.01 | 0.01 | 0.01 | 0.01 | 0.01 | 0.01 | 0.01 | 0.01 |
|  |  |  | Mannheimia | 0.01 | 0.00 | 0.00 | 0.01 | 0.01 | 0.01 | 0.01 | 0.01 | 0.01 | 0.01 | 0.00 | 0.00 | 0.01 | 0.01 | 0.01 | 0.01 |
|  |  |  | Pasteurella | 0.03 | 0.04 | 0.03 | 0.04 | 0.04 | 0.03 | 0.03 | 0.03 | 0.03 | 0.03 | 0.02 | 0.02 | 0.04 | 0.04 | 0.02 | 0.03 |
|  |  | Piscirickettsiaceae | Thiomicrospira | 0.08 | 0.08 | 0.06 | 0.05 | 0.07 | 0.09 | 0.17 | 0.14 | 0.07 | 0.09 | 0.06 | 0.05 | 0.07 | 0.08 | 0.04 | 0.11 |
|  |  | Pseudoalteromonadaceae | Pseudoalteromonas | 6.38 | 4.34 | 5.47 | 0.64 | 4.68 | 1.76 | 2.40 | 0.88 | 5.18 | 4.85 | 4.53 | 6.31 | 5.61 | 3.28 | 1.38 | 4.53 |
|  |  | Pseudomonadaceae | Azotobacter | 0.14 | 0.08 | 0.07 | 0.11 | 0.09 | 0.14 | 0.20 | 0.13 | 0.07 | 0.13 | 0.11 | 0.06 | 0.09 | 0.11 | 0.09 | 0.14 |
|  |  |  | Cellvibrio | 0.33 | 0.23 | 0.21 | 0.19 | 0.27 | 0.41 | 0.67 | 0.28 | 0.22 | 0.37 | 0.29 | 0.18 | 0.25 | 0.28 | 0.24 | 0.38 |
|  |  |  | Pseudomonas | 1.82 | 1.12 | 0.91 | 1.35 | 1.22 | 1.47 | 2.26 | 1.70 | 0.97 | 1.56 | 1.40 | 1.12 | 1.14 | 1.34 | 1.28 | 1.61 |
|  |  | Psychromonadaceae | Psychromonas | 0.03 | 0.03 | 0.03 | 0.10 | 0.03 | 0.06 | 0.05 | 0.04 | 0.04 | 0.03 | 0.05 | 0.02 | 0.03 | 0.04 | 0.03 | 0.04 |
|  |  | Shewanellaceae | Shewanella | 1.44 | 1.11 | 1.21 | 0.68 | 1.13 | 1.44 | 1.83 | 1.26 | 1.95 | 1.69 | 1.49 | 1.53 | 1.37 | 1.24 | 0.72 | 1.51 |
|  |  | unclassified (derived from Gammaproteobacteria) | Candidatus Carsonella | 0.00 | 0.00 | 0.00 | 0.00 | 0.00 | 0.00 | 0.00 | 0.00 | 0.00 | 0.00 | 0.00 | 0.00 | 0.00 | 0.00 | 0.00 | 0.00 |
|  |  |  | Congregibacter | 0.85 | 0.77 | 0.76 | 1.65 | 0.85 | 1.86 | 2.78 | 1.26 | 0.78 | 1.63 | 0.81 | 0.47 | 0.59 | 1.08 | 1.55 | 1.56 |
|  |  |  | Reinekea | 0.22 | 0.21 | 0.18 | 0.10 | 0.26 | 0.29 | 0.37 | 0.21 | 0.21 | 0.24 | 0.23 | 0.14 | 0.21 | 0.24 | 0.13 | 0.24 |
|  |  |  | unclassified (derived from Gammaproteobacteria) | 0.03 | 0.04 | 0.03 | 0.01 | 0.04 | 0.02 | 0.04 | 0.04 | 0.02 | 0.03 | 0.03 | 0.02 | 0.03 | 0.03 | 0.02 | 0.03 |
|  |  | Vibrionaceae | Aliivibrio | 0.17 | 0.16 | 0.16 | 0.08 | 0.12 | 0.33 | 0.25 | 0.16 | 1.20 | 0.22 | 0.24 | 0.16 | 0.17 | 0.32 | 0.08 | 0.16 |
|  |  |  | Listonella | 0.00 | 0.00 | 0.00 | 0.00 | 0.00 | 0.00 | 0.00 | 0.00 | 0.01 | 0.00 | 0.00 | 0.00 | 0.00 | 0.00 | 0.00 | 0.00 |
|  |  |  | Photobacterium | 0.35 | 0.27 | 0.27 | 0.21 | 0.21 | 0.51 | 0.51 | 0.33 | 1.79 | 0.39 | 0.44 | 0.21 | 0.36 | 0.49 | 0.14 | 0.27 |
|  |  |  | Vibrio | 1.22 | 1.80 | 1.81 | 0.50 | 0.83 | 4.56 | 1.81 | 1.19 | 28.70 | 3.14 | 2.63 | 1.12 | 1.06 | 4.53 | 0.46 | 1.02 |
|  |  | Xanthomonadaceae | Stenotrophomonas | 0.08 | 0.05 | 0.04 | 0.08 | 0.05 | 0.13 | 0.16 | 0.15 | 0.05 | 0.10 | 0.06 | 0.04 | 0.05 | 0.07 | 0.07 | 0.10 |
|  |  |  | Xanthomonas | 0.15 | 0.14 | 0.11 | 0.15 | 0.12 | 0.27 | 0.37 | 0.32 | 0.10 | 0.21 | 0.14 | 0.10 | 0.11 | 0.14 | 0.13 | 0.23 |
|  |  |  | Xylella | 0.03 | 0.04 | 0.03 | 0.04 | 0.03 | 0.05 | 0.07 | 0.07 | 0.03 | 0.05 | 0.04 | 0.04 | 0.03 | 0.03 | 0.04 | 0.06 |
|  | unclassified (derived from Proteobacteria) | unclassified (derived from Proteobacteria) | Magnetococcus | 0.10 | 0.08 | 0.07 | 0.06 | 0.09 | 0.14 | 0.23 | 0.23 | 0.07 | 0.13 | 0.10 | 0.06 | 0.10 | 0.08 | 0.07 | 0.13 |
|  |  |  | unclassified (derived from Proteobacteria) | 0.00 | 0.00 | 0.00 | 0.01 | 0.00 | 0.00 | 0.00 | 0.00 | 0.00 | 0.00 | 0.00 | 0.00 | 0.00 | 0.00 | 0.00 | 0.00 |
| Spirochaetes | Spirochaetia | Leptospiraceae | Leptospira | 0.06 | 0.07 | 0.06 | 0.04 | 0.08 | 0.07 | 0.13 | 0.10 | 0.05 | 0.08 | 0.05 | 0.06 | 0.06 | 0.06 | 0.05 | 0.08 |
|  |  | Spirochaetaceae | Borrelia | 0.02 | 0.01 | 0.01 | 0.01 | 0.02 | 0.01 | 0.02 | 0.01 | 0.01 | 0.01 | 0.01 | 0.01 | 0.01 | 0.01 | 0.02 | 0.02 |
|  |  |  | Treponema | 0.02 | 0.02 | 0.02 | 0.02 | 0.01 | 0.03 | 0.05 | 0.05 | 0.01 | 0.02 | 0.02 | 0.03 | 0.02 | 0.02 | 0.02 | 0.03 |
| Synergistetes | Synergistia | Synergistaceae | Dethiosulfovibrio | 0.02 | 0.02 | 0.02 | 0.02 | 0.02 | 0.03 | 0.03 | 0.04 | 0.02 | 0.03 | 0.01 | 0.01 | 0.01 | 0.02 | 0.01 | 0.02 |
|  |  |  | Thermanaerovibrio | 0.01 | 0.01 | 0.01 | 0.02 | 0.01 | 0.01 | 0.02 | 0.03 | 0.01 | 0.02 | 0.01 | 0.00 | 0.01 | 0.01 | 0.02 | 0.02 |
| Tenericutes | Mollicutes | Acholeplasmataceae | Acholeplasma | 0.00 | 0.01 | 0.01 | 0.01 | 0.01 | 0.00 | 0.01 | 0.01 | 0.00 | 0.01 | 0.00 | 0.01 | 0.00 | 0.01 | 0.01 | 0.00 |
|  |  |  | Candidatus Phytoplasma | 0.00 | 0.01 | 0.01 | 0.01 | 0.01 | 0.01 | 0.01 | 0.01 | 0.00 | 0.01 | 0.01 | 0.00 | 0.01 | 0.01 | 0.00 | 0.00 |
|  |  | Entomoplasmataceae | Mesoplasma | 0.00 | 0.00 | 0.00 | 0.00 | 0.00 | 0.00 | 0.00 | 0.00 | 0.00 | 0.00 | 0.00 | 0.00 | 0.00 | 0.00 | 0.01 | 0.00 |
|  |  | Mycoplasmataceae | Mycoplasma | 0.03 | 0.04 | 0.04 | 0.03 | 0.04 | 0.03 | 0.02 | 0.02 | 0.02 | 0.02 | 0.02 | 0.02 | 0.03 | 0.03 | 0.04 | 0.02 |
|  |  |  | Ureaplasma | 0.00 | 0.00 | 0.00 | 0.00 | 0.00 | 0.00 | 0.00 | 0.00 | 0.00 | 0.00 | 0.00 | 0.00 | 0.00 | 0.00 | 0.00 | 0.00 |
|  |  | Spiroplasmataceae | Spiroplasma | 0.00 | 0.00 | 0.00 | 0.00 | 0.00 | 0.00 | 0.00 | 0.00 | 0.00 | 0.00 | 0.00 | 0.00 | 0.00 | 0.00 | 0.00 | 0.00 |
| Thermotogae | Thermotogae (class) | Thermotogaceae | Fervidobacterium | 0.01 | 0.01 | 0.01 | 0.02 | 0.01 | 0.02 | 0.02 | 0.02 | 0.01 | 0.02 | 0.01 | 0.01 | 0.02 | 0.01 | 0.02 | 0.01 |
|  |  |  | Petrotoga | 0.01 | 0.02 | 0.02 | 0.01 | 0.02 | 0.02 | 0.02 | 0.02 | 0.01 | 0.02 | 0.01 | 0.01 | 0.02 | 0.03 | 0.03 | 0.02 |
|  |  |  | Thermosipho | 0.03 | 0.04 | 0.04 | 0.03 | 0.04 | 0.03 | 0.04 | 0.04 | 0.02 | 0.03 | 0.03 | 0.03 | 0.04 | 0.04 | 0.03 | 0.03 |
|  |  |  | Thermotoga | 0.03 | 0.05 | 0.04 | 0.04 | 0.05 | 0.05 | 0.07 | 0.09 | 0.03 | 0.05 | 0.05 | 0.01 | 0.04 | 0.04 | 0.03 | 0.05 |
| unclassified (derived from Bacteria) | unclassified (derived from Bacteria) | unclassified (derived from Bacteria) | Thermobaculum | 0.03 | 0.03 | 0.03 | 0.06 | 0.03 | 0.05 | 0.09 | 0.11 | 0.03 | 0.06 | 0.04 | 0.03 | 0.03 | 0.05 | 0.05 | 0.05 |
|  |  |  | unclassified (derived from Bacteria) | 0.00 | 0.00 | 0.00 | 0.00 | 0.00 | 0.00 | 0.00 | 0.00 | 0.00 | 0.00 | 0.00 | 0.00 | 0.00 | 0.00 | 0.00 | 0.00 |
| Verrucomicrobia | Opitutae | Opitutaceae | Opitutus | 0.11 | 0.07 | 0.07 | 0.20 | 0.07 | 0.31 | 0.53 | 0.76 | 0.11 | 0.34 | 0.11 | 0.06 | 0.07 | 0.14 | 0.09 | 0.27 |
|  | unclassified (derived from Verrucomicrobia) | Methylacidiphilaceae | Methylacidiphilum | 0.02 | 0.02 | 0.02 | 0.07 | 0.02 | 0.06 | 0.10 | 0.15 | 0.02 | 0.05 | 0.02 | 0.01 | 0.02 | 0.03 | 0.02 | 0.05 |
|  | Verrucomicrobiae | Verrucomicrobiaceae | Akkermansia | 0.05 | 0.04 | 0.04 | 0.16 | 0.04 | 0.24 | 0.25 | 0.49 | 0.05 | 0.17 | 0.06 | 0.03 | 0.04 | 0.06 | 0.06 | 0.18 |
