# Supplementary figures and images for: Aura-biomes are present in the water layer above coral reef benthic macro-organisms
Source: PeerJ. 2017 Aug 15;5:e3666. doi: 10.7717/peerj.3666 (PMC5562181; doi:10.7717/peerj.3666)

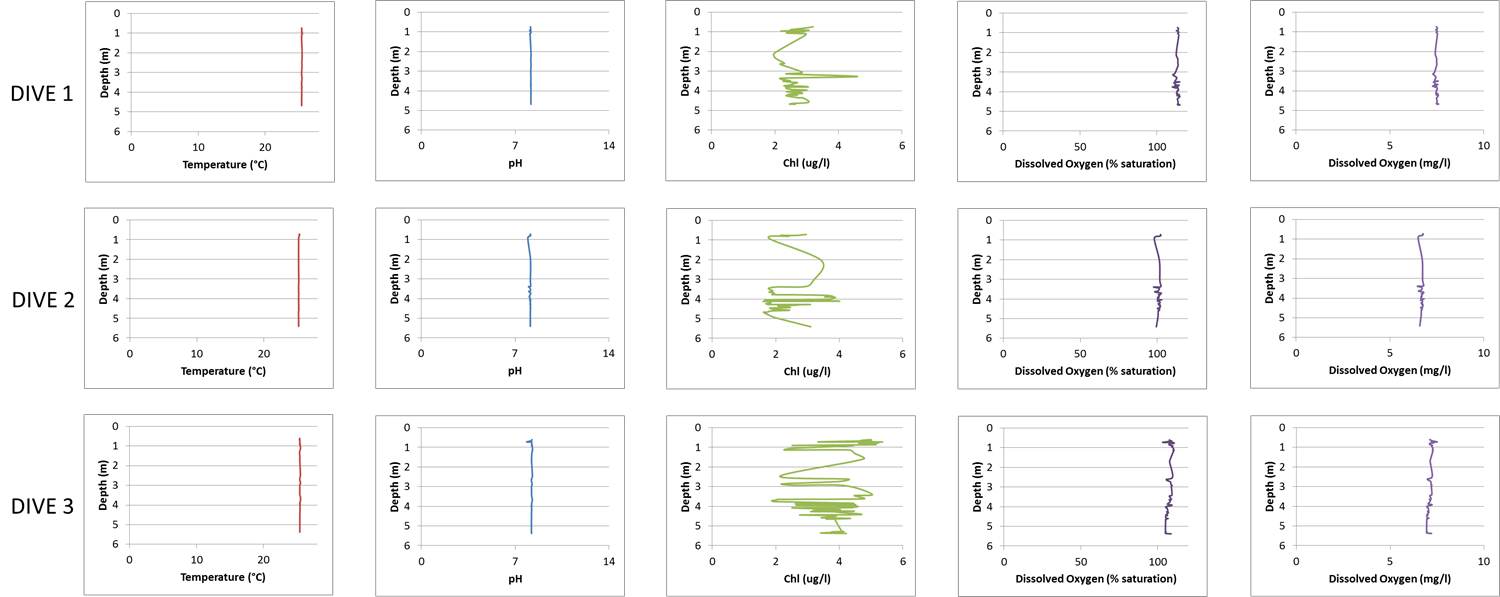

Supplement: Figure S1 — Water conditions were recorded for each dive with the Manta2 Series Multiprobe TM. [file peerj-05-3666-s004.png]

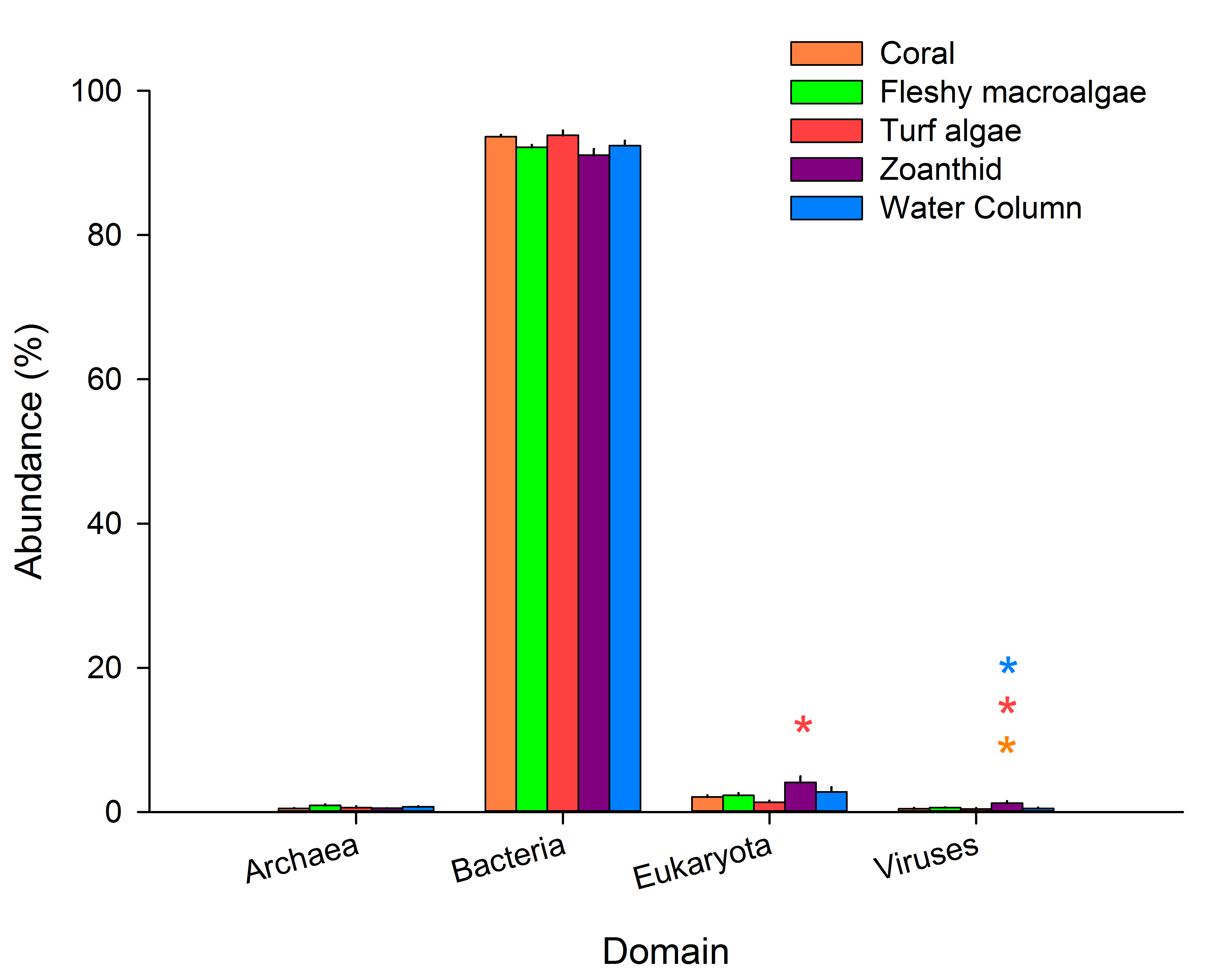

Supplement: Figure S2 — The average abundances of sequences at the domain level were compared between macro-organism aura-biomes. Asterisks above a domain designate a significance difference, while color delineates which samples varied. [file peerj-05-3666-s005.png]

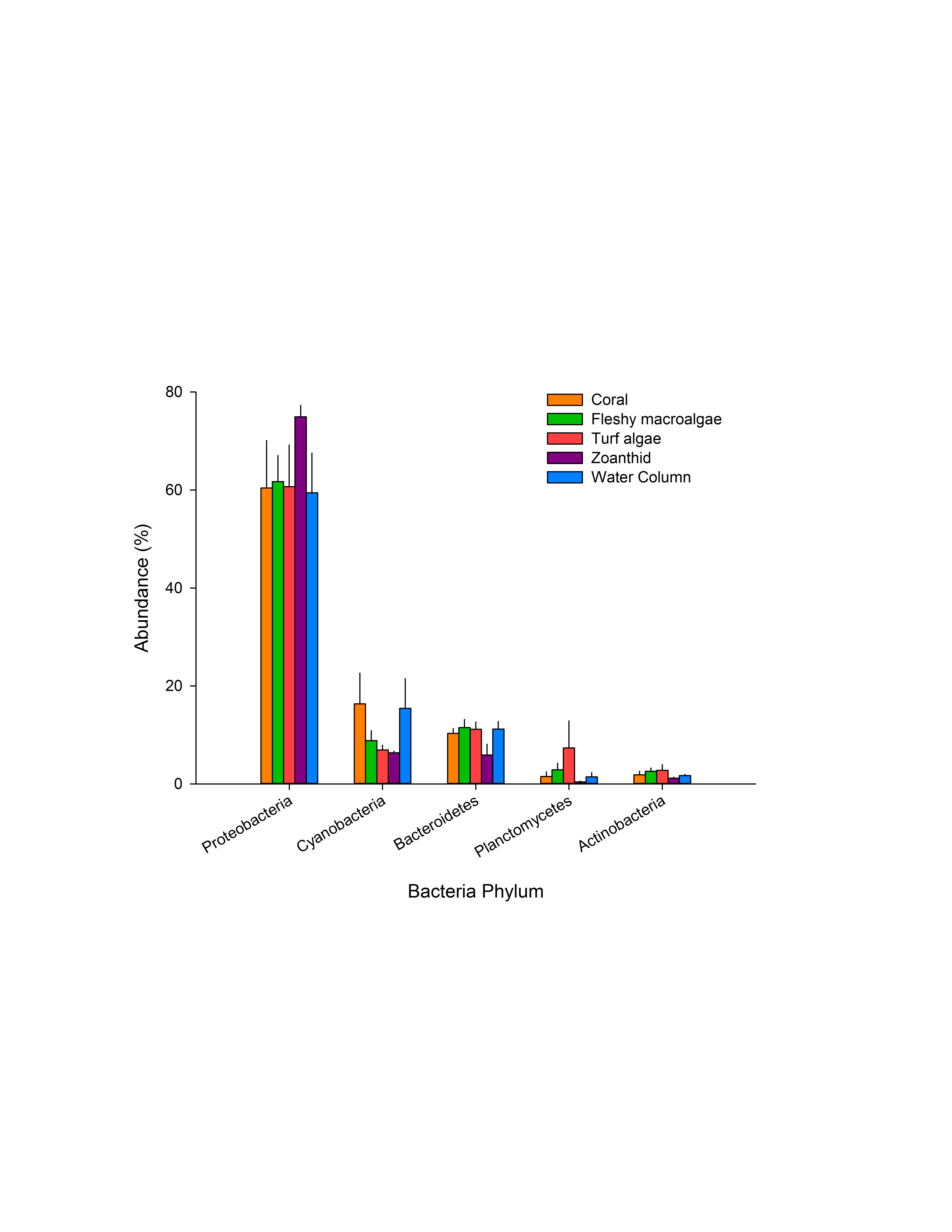

Supplement: Figure S3 — The average abundances of sequences at the phyla level within the domain Bacteria were compared between macro-organism aura-biomes. Asterisks above a domain designate a significance difference, while color delineates which samples varied. [file peerj-05-3666-s006.png]
